# Synthesis of 2,5-Dialkyl-1,3,4-oxadiazoles Bearing Carboxymethylamino Groups

**DOI:** 10.3390/molecules27227687

**Published:** 2022-11-09

**Authors:** Marcin Łuczyński, Kornelia Kubiesa, Agnieszka Kudelko

**Affiliations:** Department of Chemical Organic Technology and Petrochemistry, The Silesian University of Technology, Krzywoustego 4, PL-44100 Gliwice, Poland

**Keywords:** 1,3,4-oxadiazoles, organic ligands, heterocycles, substitution, diisopropyl iminodiacetate

## Abstract

A series of new symmetrical 2,5-dialkyl-1,3,4-oxadiazoles containing substituted alkyl groups at the terminal positions with substituents, such as bromine, isopropyloxycarbonylmethylamino, and carboxymethylamino, were successfully synthesized. The developed multistep method employed commercially available acid chlorides differing in alkyl chain length and terminal substituent, hydrazine hydrate, and phosphorus oxychloride. The intermediate bromine-containing 2,5-dialkyl-1,3,4-oxadiazoles were easily substituted with diisopropyl iminodiacetate, followed by hydrolysis in aqueous methanol solution giving the corresponding 1,3,4-oxadiazoles bearing carboxymethylamino substituents. The structure of all products was confirmed by conventional spectroscopic methods including ^1^H NMR, ^13^C NMR, and HRMS.

## 1. Introduction

Oxadiazoles are five-membered heterocyclic compounds composed of two nitrogen atoms and one oxygen atom. Depending on the heteroatom position, oxadiazoles exist in the form of several isomers [1], including 1,3,4-oxadiazole derivatives that are the most studied due to their high stability and wide range of biological activity [2]. Additionally, they exhibit anti-inflammatory, analgesic [3], antiviral [4], antibacterial [5], antifungal [6], anticancer [7], and blood pressure-lowering effects [8]. Reports have shown that oxadiazoles biological activity characteristics can be employed in agriculture as herbicides, insecticides, and plant protection agents against bacteria, viruses, and fungi [9,10,11,12]. Furthermore, oxadiazole compounds possess valuable optical properties. 1,2-Diazole fragment of 1,3,4-oxadiazole derivatives has an electron-accepting effect and contributes to its application in various types of conducting systems, such as organic light-emitting diodes, laser dyes, optical brighteners, and scintillators [13,14,15,16]. Certain 2,5-disubstituted 1,3,4-oxadiazole derivatives have high thermal and chemical stability, which is important in materials science, and such compounds are used in the production of heat-resistant polymers, blowing agents, optical brighteners, and anti-corrosion agents [17,18,19,20].

One of the most popular methods for 1,3,4-oxadiazole derivatives preparation include the cyclodehydration reaction. The following reagents are commonly employed in the cyclization of *N*,*N*′-diacylhydrazines: polyphosphoric acid (PPA) [21], sulfuric acid (H_2_SO_4_) [22], phosphorus oxychloride (POCl_3_) [23], thionyl chloride (SOCl_2_) [24], trifluoromethanesulfonic anhydride ((CF_3_SO_2_)_2_O) [25], phosphorus pentoxide (P_2_O_5_) [26], boron trifluoride etherate (BF_3_∙OEt_2_) [27], and Burgess reagent [28]. Additionally, it has been shown that the preparation of 1,3,4-oxadiazole derivatives is possible using oxidative cyclization of *N*-acylhydrazones with oxidizing agents such as cerium ammonium nitrate (CAN) [29], bromine (Br_2_) [30], potassium permanganate (KMnO_4_) [31], lead(IV) oxide (PbO_2_) [32], chloramine T [33], 2,3-dichloro-5,6-dicyano-1,4-benzowuinone (DDQ) [34], and hypervalent iodine reagents [35].

1,3,4-Oxadiazoles containing alkyl chains at the 2 and 5 positions and substituted with carboxymethylamino groups are of particular interest. Generally, the presence of aminopolycarboxylic functionalities promote complexing properties in such derivatives, allowing for binding to metal cations. The literature shows a range of organic ligands of this type, some of which have been approved for use in medicine and agriculture. These include ethylenediaminetetraacetic acid (EDTA) [36], *N*-(hydroxyethyl)ethylenediaminetriacetic acid (HEEDTA) [37], ethylenediamine-*N*,*N*′-bis(*o*-hydroxy-*p*-methylphenyl)acetic acid (EDDHMA) [38], diethylenetriaminepentaacetic acid (DTPA) [39], nitrilotriacetic acid (NTA) [40], glucoheptanoic acid [41], and citric acid [42,43]. Unfortunately, despite the excellent chelating properties, not all fertilizing chelates show the adequate biodegradation. Some of the most common coordination compounds based on EDTA and DTPA are characterized by very high stability, but they are resistant to biodegradation. Numerous studies revealed that they are present in waters of lakes or ponds, as well as in the soil for a long time, which may result in eutrophication of waters and introduction of metals into the food chain [44]. On the other hand, there has recently been an emphasis on chelating agents based on sugar molecules in order to increase the effectiveness of the micronutrients. There is a possibility that sugar acid derivatives, condensed tannis, and glucohetonates could effectively replace the traditionally used EDTA for the production of chelated micronutrients [43]. Heterocyclic compounds composed of carbon, oxygen, and nitrogen atoms could also constitute another alternative scaffold for the construction of new chelating agents.

**Scheme 1:**
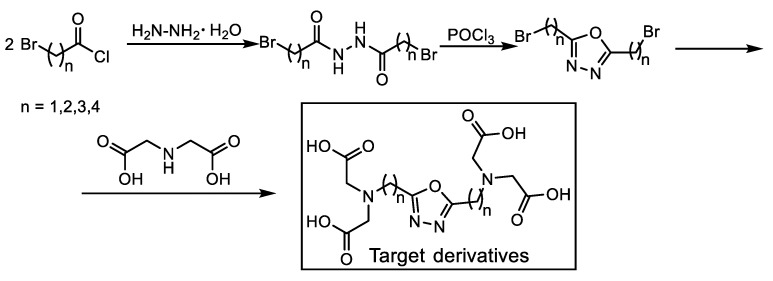
Initial concept of the synthetic pathway.

According to the above data, we assumed that the combination of the 1,3,4-oxadiazole core containing oxygen and two nitrogen atoms in the ring with aminopolycarboxylic groups allows for the development of a new family of organic ligands with potential applications as complexing agents in medicine and agriculture. Herein, we developed an effective method for the preparation of new symmetrically substituted 1,3,4-oxadiazole derivatives containing carboxymethylaminoalkyl groups at the 2 and 5 positions. Initially, a three-step transformation was conducted using commercially available reagents (Scheme 1). In the first step, the acid chlorides were reacted with hydrazine hydrate to form symmetrical *N*,*N*′-diacylhydrazine derivatives with different alkyl chain lengths. In the second step, they were cyclized using POCl_3_, a known cyclodehydration reagent. Finally, the substitution reaction between bromine-containing 1,3,4-oxadiazole derivatives and iminodiacetic acid was studied.

## 2. Results

**Scheme 2:**
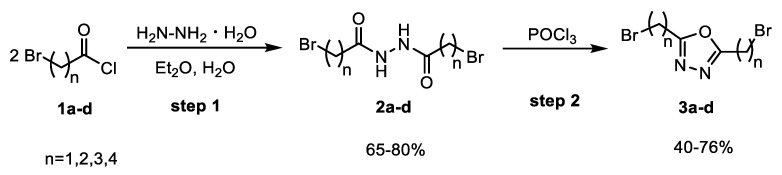
Synthesis of symmetrical *N*,*N*′-diacylhydrazine (**2a**–**d**) and 1,3,4-oxadiazole derivatives (**3a**–**d**). Reaction conditions: step 1: acid chloride (**1a**–**d**, 0.06 mol), hydrazine hydrate (hydrazine 64%, 4.6 mL, 0.06 mol), sodium carbonate (6.36 g, 0.06 mol), diethyl ether (70 mL), water (40 mL), 0 °C, 0.5 h; acid chloride (**1a**–**d**, 0.06 mol), diethyl ether (10 mL), 25 °C, 2 h; step 2: *N*,*N*′-diacylhydrazine (**2a**–**d**, 0.007 mol), POCl_3_ (22.4 mL, 0.24 mol), 55 °C, 6–24 h.

The target derivatives containing carboxymethylaminoalkyl groups were obtained in a multistep transformation reaction. The first step consisted of the synthesis of symmetrical *N*,*N*′-diacylhydrazine derivatives (**2a**–**d**) (Scheme 2) using commercially available acid chloride derivatives (**1a**–**d**) (Scheme 2) differing in alkyl chain length and bearing a bromine atom at the terminal position. The model reaction employed bromoacetyl chloride (**1a**) as the starting material. The optimization study consisted of examining the base (triethylamine (TEA) and sodium carbonate), solvent (chloroform, and diethyl ether), and the influence of temperature. The best results were obtained at low temperature (0 °C) in diethyl ether using aqueous sodium carbonate as the base. The products were purified via recrystallization from either methanol or ethanol. The yields of the obtained hydrazine derivatives (**2a**–**d**) were 65–79% (Table 1, entries 4, 8, 12, and 16).

The synthesis of 1,3,4-oxadiazole derivatives (**3a**–**d**) (Scheme 2) involved reacting with POCl_3_, a cyclodehydration reagent. The reaction was conducted in anhydrous toluene or solvent-free conditions and monitored by TLC. In toluene, product was formed in lower yield compared to the solvent-free reaction (Table 2, entries 2, 4, 6, and 8). The final products (**3a**–**d**) were obtained in 40–76% yield and were used for the subsequent reactions without purification (Table 2).

**Scheme 3:**
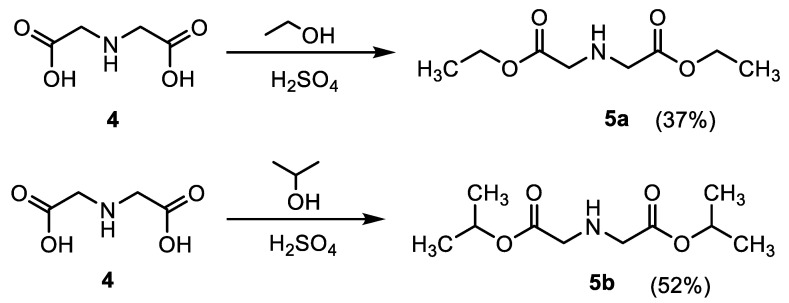
Synthesis of diethyl iminodiacetate (**5a**) and diisopropyl iminodiacetate (**5b**). Reaction conditions: iminodiacetic acid (**4**, 15.0 g, 0.11 mol), alcohol (120 mL), H_2_SO_4_ (7.5 mL), reflux, 12 h.

As part of the synthesis, we planned to use iminodiacetic acid (**4**), which could directly react with the formed 2,5-dialkyl-1,3,4-oxadiazole derivatives (**3a**–**d**) containing bromine substituents (Scheme 1). After testing a series of bases, including TEA, sodium hydroxide, and sodium carbonate, as well as several organic solvents (chloroform, methanol, acetonitrile) and their mixtures [45], the desired products **7** were not generated. Therefore, the iminodiacetic acid was converted into a more reactive ester, which could be subjected to a substitution reaction with 2,5-bis(bromoalkyl)-1,3,4-oxadiazoles (**3a**–**d**), followed by hydrolysis to restore the carboxyl groups. Hence, the esterification reactions were performed using ethanol (**a**) and isopropanol (**b**) as substrates, and in the presence of sulfuric acid as the catalyst (Scheme 3). The final esters (**5a**,**b**) were obtained in good yields (37–52%).

**Scheme 4:**
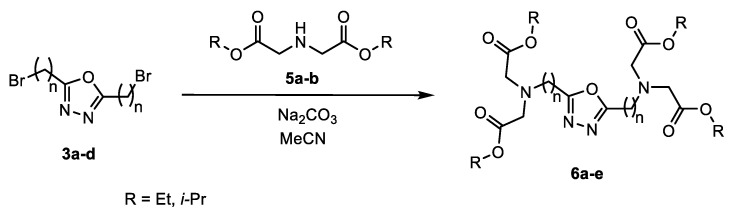
Synthesis of 2,5-dialkyl-1,3,4-oxadiazole (**6a**–**e**) ester derivatives. Reaction conditions: 1,3,4-oxadiazole derivative (**3a**–**d**, 0.004 mol), iminodiacetic acid ester (**5a**,**b**, 0.01 mol), sodium carbonate (4.24 g, 0.04 mol), acetonitrile (50 mL), 60 °C, 12 h.

The next step was to perform the substitution reaction involving the appropriate ester (**5a**,**b**), oxadiazole derivative (**3a**–**d**), and base in an aprotic solvent (Scheme 4). In order to determine the optimal conditions, 2,5-bis(bromomethyl)-1,3,4-oxadiazole (**3a**) and diethyl iminodiacetate (**5a**) were first examined. When the reaction was conducted at room temperature, main product **6a** was produced but in a low yield, at 23% (Table 3, entry 11). The further study revealed that the optimal temperature was in the range of 50–60 °C, giving the product **6a** in a 91% yield (Table 3, entry 12).

Among the solvents tested, acetonitrile gave the best result (Table 3, entry 12) owing to its characteristic aprotic polarity and its relatively low boiling point in relation to dimethylformamide (DMF) or DMSO. The inorganic weak base sodium carbonate enhanced the removal of inorganic compounds during the extraction process. Additionally, the optimal reaction time was 8–12 h, which was determined by TLC. Having the optimized conditions in hand, a series of substitution reactions were conducted using oxadiazoles (**3b**–**d**), and producing products (**6a**–**e**) in a 71–91% yield (Table 4, entries 1–5). All products (**6a**–**e**) were purified by column chromatography on silica gel using ethyl acetate as the eluent.

**Scheme 5:**
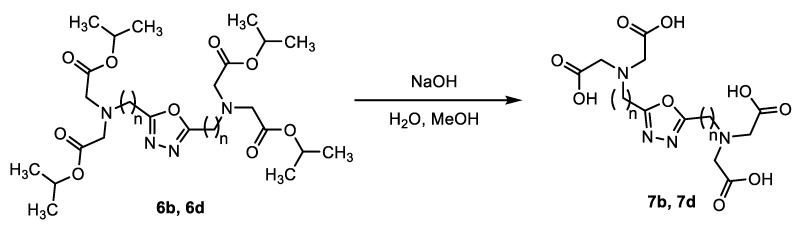
Hydrolysis reaction of compounds **6b** and **6d** to form 2,5-dialkyl-1,3,4-oxadiazole derivatives containing carboxymethylaminoalkyl moieties (**7b**, **7d**). Reaction conditions: ester derivative of 2,5-dialkyl-1,3,4-oxadiazole (**6b**, **6d**, 0.18 mmol), NaOH (0.1 g, 1.8 mmol), MeOH (24 mL), H_2_O (6 mL), 50 °C, 1 h.

The last step in the synthetic pathways was the restoration of the carboxyl groups from esters groups (**6**). Various literature methods were examined, including anhydrous lithium chloride [46] and the classical method of hydrolysis in an acidic and alkaline environment [47]. It was found that the alkaline hydrolysis reaction gave desired 1,3,4-oxadiazole derivatives bearing carboxymethylaminoalkyl groups (**7b**, **7d**) at the side alkyl chains (Scheme 5). The lithium chloride method was ineffective, and hydrolysis under acidic conditions gave the desired product, but excessive heating promoted decomposition of the oxadiazole. Optimization of the hydrolysis reaction involved different amounts of NaOH and solvents. The obtained results showed that a significant excess of NaOH and conducting the reaction in a relatively high temperature caused the decomposition. However, we observed the formation of the intended final product (**7b**) when the molar ratio between ester **6** and NaOH was 1:10. Methanol was found to be the best solvent owing to its ability to dissolve the substrate. The final products (**7b**, **7d**) were purified by recrystallization from methanol, providing the pure products in a 54–68% yield (Table 4, entries 6 and 7).

The structure of all obtained intermediates and final products was confirmed by ^1^H and ^13^C NMR spectroscopy (see Supplementary Materials. Both ester and acid derivatives were symmetrical molecules; hence, the number of signals was reduced. Among 2,5-dialkyl-1,3,4-oxadiazole derivatives (**6a**–**e**), and not described so far in the literature, characteristic ^1^H NMR signals included the doublet at 1.25 ppm and septet at 5.00 ppm corresponding to the isopropyl group. The singlet at 3.50 ppm was related to the iminodiacetate moiety. The remaining signals at 1.00–3.00 ppm corresponded to the alkyl side chain between the oxadiazole and diisopropyl ester. In the case of ^13^C NMR, the characteristic C2 and C5 signals of the heterocyclic 1,3,4-oxadiazole ring were found at 165.0 ppm. The remaining peaks from carbonyl groups were located at 170.0 ppm, while two signals from isopropyloxy group were found at 22.0 ppm (CH_3_) and 68.0 ppm (-CH-), respectively. Signals at 50.0–55.0 ppm corresponded to the iminodiacetate part of the molecule (-N(CH_2_)_2_<). Finally, carbons of the alkyl chain were in the range of 22.0–60.0 ppm. ^1^H and ^13^C spectra of the final products, containing carboxyl groups (**7b**, **7d**), showed no visible signals of the ester residue. High-resolution mass spectra further confirmed the structure of the obtained intermediates and final products.

## 3. Experimental Section

### 3.1. General Information

All reagents were purchased from commercial sources and used without further purification. Melting points were measured using a Stuart SMP3 melting point apparatus (Staffordshire, UK). NMR spectra were recorded at 25 °C using an Agilent 400-NMR spectrometer (Agilent Technologies, Waldbronn, Germany) at 400 MHz for ^1^H and 100 MHz for ^13^C, with CDCl_3_ or DMSO as solvent, and TMS as the internal standard. High-resolution mass spectra were acquired using a Waters ACQUITY UPLC/Xevo G2QT instrument (Waters Corporation, Milford, MA, USA). Thin-layer chromatography (TLC) was performed using silica gel 60 F254 (Merck, Merck KGaA, Darmstadt, Germany) thin-layer chromatography plates, with ethyl acetate, chloroform/ethyl acetate (5:1 *v*/*v*), or methanol/chloroform (4:1 *v*/*v*) as the mobile phases.

### 3.2. Synthesis and Characterization

#### 3.2.1. Synthesis of N,N′-Diacylhydrazine Derivatives (**2a**–**d**)

Hydrazine hydrate (4.6 mL, 0.06 mol) was dissolved in diethyl ether (50 mL), and the mixture was cooled to 0 °C. The appropriate amount of acid chloride (**1a**–**d**, 0.06 mol) was dissolved in diethyl ether (20 mL) and added dropwise to the mixture. The temperature was carefully monitored, keeping it below 35 °C. Then, after 30 min, sodium carbonate (6.36 g, 0.06 mol) dissolved in water (40 mL) was added. After the evolution of carbon dioxide had ceased, acid chloride (**1a**–**d**, 0.06 mol) dissolved in diethyl ether (10 mL) was added dropwise. The reaction mixture was stirred at room temperature for 2 h. The resulting precipitate was filtered, and dried products was recrystallized from methanol to obtain the desired pure products.

2-Bromo-*N*′-(2-bromoacetyl)acetohydrazide (**2a**)

The product was obtained as white powder (10.69 g, 65%); m.p. 174–176 °C. ^1^H-NMR (400 MHz, DMSO): δ 3.92 (s, 4H), 10.58 (s, 2H, NH); ^13^C-NMR (100 MHz, DMSO): δ 26.9, 164.4.

3-Bromo-*N*′-(3-bromopropanoyl)propanehydrazide (**2b**)

The product was obtained as white powder (13.23 g, 73%); m.p. 182–183 °C. ^1^H-NMR (400 MHz, DMSO): δ 2.78(t, *J* = 8.0 Hz, 4H), 3.65 (t, *J* = 8.0 Hz, 4H), 10.08 (s, 2H, NH);); ^13^C-NMR (100 MHz, DMSO): δ 28.7, 36.3, 167.7.

4-Bromo-*N*′-(4-bromobutanoyl)butanehydrazide (**2c**)

The product was obtained as white powder (15.05 g, 76%); m.p. 159–160 °C. ^1^H-NMR (400 MHz, DMSO): δ 2.04 (tt, *J* = 6,8 Hz, *J* = 7.2 Hz, 4H), 2.28 (t, *J* = 7.2 Hz, 4H), 3.55 (t, *J* = 6.8 Hz, 4H), 9.75 (s, 2H, NH);); ^13^C-NMR (100 MHz, DMSO): δ 28.3, 31.5, 34.2, 170.1.

5-Bromo-*N*′-(5-bromopentanoyl)pentanehydrazide (**2d**)

The product was obtained as white powder (16.97 g, 79%); m.p. 149–151 °C. ^1^H-NMR (400 MHz, DMSO): δ 1.61 (m, 4H), 1.81 (m, 4H), 2.13 (t, J = 8.0 Hz, 4H), 3.52 (t, J = 8.0 Hz, 4H), 9.67 (s, 2H, NH); ^13^C-NMR (100 MHz, DMSO): δ 23.6, 31.5, 32.1, 34.7, 170.7.

#### 3.2.2. Synthesis of 1,3,4-Oxadiazole Derivatives (**3a**–**d**)

Phosphorus oxychloride (22.4 mL, 0.24 mol) was added to *N*,*N*′-diacylhydrazine (**2a-d**, 0.007 mol). The mixture was heated to reflux for 6–24 h. The progress of the reaction was monitored by TLC using methanol/chloroform (4:1 *v*/*v*) as the mobile phase. Excess phosphorus oxychloride was evaporated, and the residue in the flask was dissolved in diethyl ether (40 mL) and poured into water (100 mL). The mixture was neutralized using sodium carbonate, extracted with diethyl ether (40 mL), dried over anhydrous magnesium sulfate, and evaporated to dryness.

2,5-Bis(bromomethyl)-1,3,4-oxadiazole (**3a**)

The product was obtained as yellow oil (0.91 g, 51%). ^1^H-NMR (400 MHz, CDCl_3_): δ 4.92 (s, 4H); ^13^C-NMR (100 MHz, DMSO): δ 17.6, 164.2. HRMS (ESI): *m/z* calcd for C_4_H_4_N_2_OBr_2_ + H^+^: 256.8748; found 256.8756.

2,5-Bis(2-bromoethyl)-1,3,4-oxadiazole (**3b**)

The product was obtained as yellow oil (1.99 g, 40%). ^1^H-NMR (400 MHz, DMSO): δ 2.96 (t, *J* = 8.0Hz, 4H), 3.56 (t, *J* = 8.0 Hz, 4H); ^13^C-NMR (100 MHz, DMSO): δ 28.7, 36.3, 167.7. HRMS (ESI): *m/z* calcd for C_6_H_8_N_2_OBr_2_ + H^+^: 284.9061; found 284.9064.

2,5-Bis(3-bromopropyl)-1,3,4-oxadiazole (**3c**)

The product was obtained as yellow oil (0.96 g, 44%). ^1^H-NMR (400 MHz, CDCl_3_): δ 2.21 (m, 4H), 2.96 (t, *J* = 8.0 Hz, 4H), 3.63 (t, *J* = 8.0 Hz, 4H); ^13^C-NMR (100 MHz, DMSO): δ 28.9, 31.5, 33.5, 165.5. HRMS (ESI): *m/z* calcd for C_8_H_12_N_2_OBr_2_ + H^+^: 312.9374; found 312.9386.

2,5-Bis(4-bromobutyl)-1,3,4-oxadiazole (**3d**)

The product was obtained as yellow oil (1.81 g, 76%). ^1^H-NMR (400 MHz, DMSO): δ 1.77 (m, 4H), 1.83 (m, 4H), 2.81 (t, *J* = 8.0 Hz, 4H), 3.53 (t, *J* = 8.0 Hz, 4H); ^13^C-NMR (100 MHz, DMSO): δ 23.6, 24.4, 31.4, 34.4, 166.1. HRMS (ESI): *m/z* calcd for C_10_H_16_N_2_OBr_2_ + H^+^: 340.9688; found 340.9692.

#### 3.2.3. Synthesis of Iminodiacetic Acid Ester Derivatives (**5a**,**b**)

Ethanol (**a**) or isopropanol (**b**) (120 mL) and concentrated H_2_SO_4_ (7.5 mL) were added to iminodiacetic acid (**4**) (15.0 g, 0.11 mol). The reaction mixture was heated to reflux for 12 h. Excess alcohol was then evaporated using a rotary evaporator, and the mixture was neutralized with sodium bicarbonate solution. Then, the resulted solution was extracted with ethyl acetate (30 mL), dried over anhydrous magnesium sulfate, and evaporated to dryness.

Diethyl iminodiacetate (**5a**)

The product was obtained as slightly yellow liquid (7.69 g, 37%). ^1^H-NMR (400 MHz, CDCl_3_): δ 1.24 (t, *J* = 8.0 Hz, 6H), 3.42 (s, 4H), 4.15 (m, 4H); ^13^C-NMR (100 MHz, DMSO): δ 14.1, 50.1, 60.8, 171.6. HRMS (ESI): *m/z* calcd for C_8_H_15_NO_4_ + H^+^: 190.1079; found 190.1082 [48,49].

Diisopropyl iminodiacetate (**5b**)

The product was obtained as slightly yellow liquid (12.41 g, 52%). ^1^H-NMR (400 MHz, CDCl_3_): δ 1.25 (d, *J* = 6.4 Hz, 12H), 3.42 (s, 4H), 5.07 (m, 2H); ^13^C-NMR (100 MHz, CDCl_3_): δ 21.5, 50.3, 68.4, 171.2. HRMS (ESI): *m/z* calcd for C_10_H_19_NO_4_ + H^+^: 218.1392; found 218.1403.

#### 3.2.4. Synthesis of Ester Derivatives of 2,5-Dialkyl-1,3,4-oxadiazole (**6a**–**e**)

2,5-Bis(bromoalkyl)-1,3,4-oxadiazole (**3a**–**d**, 0.004 mol), iminodiacetic acid ester (**5a**–**b**, 0.01 mol), and sodium carbonate (4.24 g, 0.04 mol) were dissolved in acetonitrile (50 mL). The reaction mixture was heated at 60 °C for 12 h. Water (20 mL) was added and extracted using ethyl acetate (50 mL). The organic phase was separated, dried over anhydrous magnesium sulfate, and evaporated to dryness. The crude product was purified using column chromatography with ethyl acetate as the mobile phase.

Tetraethyl 2,2′,2″,2‴-(((1,3,4-oxadiazole-2,5-diyl)bis(methylene))bis(azanetriyl))tetraacetate (**6a**)

The product was obtained as yellow oil (1.72 g, 91%). ^1^H-NMR (400 MHz, CDCl_3_): δ 1.26 (t, *J* = 8.0 Hz, 12H), 3.69 (s, 8H), 4.24 (q, *J* = 8.0 Hz, 8H); ^13^C-NMR (100 MHz, CDCl_3_): δ 14.1, 48.1, 54.5, 60.8, 164.3, 170.5. HRMS (ESI): *m/z* calcd for C_20_H_32_N_4_O_9_ + H^+^: 473.2248; found 473.2253.

Tetraisopropyl 2,2′,2″,2‴-(((1,3,4-oxadiazole=2,5-diyl)bis(methylene))bis(azanetriyl))tetraacetate (**6b**)

The product was obtained as yellow oil (1.77 g, 84%). ^1^H-NMR (400 MHz, CDCl_3_): δ 1.24 (d, *J* = 6.4 Hz, 24H), 3.65 (s, 8H), 4.24 (s, 4H), 5.02 (sept, *J* = 6.4 Hz, 4H); ^13^C-NMR (100 MHz, CDCl_3_): δ 21.8, 48.1, 54.8, 68.4, 164.4, 170.1. HRMS (ESI): *m/z* calcd for C_24_H_40_N_4_O_9_ + H^+^: 529.2874; found 529.2870.

Tetraisopropyl 2,2′,2″,2‴-(((1,3,4-oxadiazole-2,5-diyl)bis(ethane-2,1-diyl))bis(azanetriyl))tetraacete (**6c**)

The product was obtained as yellow oil (1.58 g, 71%). ^1^H-NMR (400 MHz, CDCl_3_): δ 1.25 (d, *J* = 4.4 Hz, 24H), 3.34 (t, *J* = 4.4 Hz, 4H), 3.43 (s, 8H), 3.90 (t, *J* = 4.4 Hz, 4H), 5.07 (sept, *J* = 4.4 Hz, 4H); ^13^C-NMR (100 MHz, CDCl_3_): δ 21.8, 29.0, 39.4, 50.3, 68.4, 164.1, 171.2. HRMS (ESI): *m/z* calcd for C_26_H_44_N_4_O_9_ + H^+^: 557.3185; found 557.3185.

Tetraisopropyl 2,2′,2″,2‴-(((1,3,4-oxadiazole-2,5-diyl)bis(propane-3,1-diyl))bis(azanetriyl))tetraacetate (**6d**)

The product was obtained as yellow oil (1.59 g, 68%). %). ^1^H-NMR (400 MHz, CDCl_3_): δ 1.24 (d, *J* = 4.4 Hz, 24H), 1.94 (m, 4H), 2.84 (t, *J* = 4.4 Hz, 4H), 2.91 (t, *J* = 4.8 Hz, 4H), 3.50 (s, 8H), 5.03 (m, 4H); ^13^C-NMR (100 MHz, CDCl_3_): δ 21.9, 22.8, 24.7, 53.1, 55.2, 68.0, 166.8, 170.6. HRMS (ESI): *m/z* calcd for C_28_H_48_N_4_O_9_ + H^+^: 585.3500; found 585.3491.

Tetraisopropyl 2,2′,2″,2‴-(((1,3,4-oxadiazole-2,5-diyl)bis(butane-4,1-diyl))bis(azanetriyl))tetraacetate (**6e**)

The product was obtained as yellow oil (1.79 g, 73%). %). ^1^H-NMR (400 MHz, CDCl_3_): δ 1.26 (d, *J* = 8.0 Hz, 24H), 1.36 (m, 4H), 1.58 (m, 4H), 2.77 (t, *J* = 8.0 Hz, 4H), 2.84 (t, *J* = 8.0 Hz, 4H), 3.51 (s, 8H), 5.01 (m, 4H); ^13^C-NMR (100 MHz, CDCl_3_): δ 21.7, 23.6, 23.9, 25.1, 49.1, 54.8, 68.4, 164.4, 170.1. HRMS (ESI): *m/z* calcd for C_30_H_52_N_4_O_9_ + H^+^: 613.3812; found 613.3810.

#### 3.2.5. Synthesis of 2,5-Dialkyl-1,3,4-oxadiazole Derivatives Containing Carboxymethylamino Groups (**7b**, **7d**)

The ester derivatives of 2,5-dialkyl-1,3,4-oxadiazole (**6b**, **6d**) (0.18 mmol) were dissolved in methanol (24 mL), water (6 mL), and NaOH (0.1 g, 1.8 mmol). The reaction mixture was heated at 50 °C for 1 h. The solution was then neutralized with 1 M HCl and evaporated to dryness. The crude product was purified by recrystallization from methanol.

2,2′,2″,2‴-(((1,3,4-oxadiazole-2,5-diyl)bis(methylene))bis(azanetriyl))tetraacetic acid (**7b**)

The product was obtained as white powder (0.04 g, 54%). ^1^H-NMR (400 MHz, DMSO): δ 3.38 (s, 8H), 4.04 (s, 4H); ^13^C-NMR (100 MHz, DMSO): δ 47.4, 57.5, 164.5, 173.9. HRMS (ESI): *m/z* calcd for C_12_H_16_N_4_O_9_ + H^+^ + Na: 384.0893; found 384.0846.

2,2′,2″,2‴-(((1,3,4-oxadiazole-2,5-diyl)bis(propane-3,1-diyl))bis(azanetriyl))tetraacetic acid (**7d**)

The product was obtained as white powder (0.05 g, 68%). ^1^H-NMR (400 MHz, DMSO): δ 1.79 (m, 4H), 2.71 (t, J = 8.0 Hz, 4H), 2.83 (t, J = 8.0 Hz, 4H), 3.42 (s, 8H); ^13^C-NMR (100 MHz, DMSO): δ 22.1, 24.1, 52.8, 54.7, 166.3, 172.4. HRMS (ESI): *m/z* calcd for C_16_H_24_N_4_O_9_ + H^+^: 417.1621; found 417.1638.

## 4. Conclusions

An interesting methodological process was developed for the synthesis of extended 1,3,4-oxadiazole derivatives containing carboxymethylaminoalkyl groups at positions 2 and 5. The initial synthetic pathway, comprised of nucleophilic substitution of bromine-containing 2,5-dialkyl-1,3,4-oxadiazoles with iminodiacetic acid, was ineffective. However, the replacement of iminodiacetic acid with more reactive diisopropyl iminodiacetate led to the formation of the intermediate esters and final acids in satisfactory yields. The obtained final products constitute a new family of complexing agents with potential applications in various areas, such as agriculture, medicine, or pharmacy.

## Figures and Tables

**Table 1:** *N*,*N*′-Diacylhydrazines (**2a**–**d**) derived from acid chlorides.

Entry	Product	n	Base	Solvent	Yield [%]
1	**2a**	1	TEA	Chloroform	26
2				Diethyl ether	11
3			Na_2_CO_3_	Chloroform	47
4				Diethyl ether	65
5	**2b**	2	TEA	Chloroform	33
6				Diethyl ether	44
7			Na_2_CO_3_	Chloroform	59
8				Diethyl ether	73
9	**2c**	3	TEA	Chloroform	37
10				Diethyl ether	35
11			Na_2_CO_3_	Chloroform	66
12				Diethyl ether	76
13	**2d**	4	TEA	Chloroform	28
14				Diethyl ether	20
15			Na_2_CO_3_	Chloroform	74
16				Diethyl ether	79

**Table 2:** 2,5-Dialkyl-1,3,4-oxadiazole derivatives (**3a**–**d**) formed by cyclization of *N*,*N*′-diacylhydrazine.

Entry	Product	n	Solvent	Yield (%)
1	**3a**	1	Toluene	36
2			-	51
3	**3b**	2	Toluene	15
4			-	40
5	**3c**	3	Toluene	39
6			-	44
7	**3d**	4	Toluene	59
8			-	76

**Table 3:** Optimization of the substitution reaction with diethyl iminodiacetate to form derivative **6a**.

Entry	Solvent	Temp. (°C)	Base	Yield (%)
1	DMF	25	TEA	18
2		60		22
3		25	Na_2_CO_3_	14
4		60		69
5	DMSO	25	TEA	9
6		60		24
7		25	Na_2_CO_3_	18
8		60		55
9	Acetonitrile	25	TEA	21
10		60		39
11		25	Na_2_CO_3_	23
12		60		91

**Table 4:** Obtained yields of 2,5-dialkyl-1,3,4-oxadiazole (**6a**–**e**) and products of their hydrolysis containing carboxylic groups (**7b**,**7d**).

Entry	n	R	Product	Yield (%)
1	1	Ethyl	**6a**	91
2	1	*i-*Propyl	**6b**	84
3	2	*i-*Propyl	**6c**	71
4	3	*i-*Propyl	**6d**	68
5	4	*i-*Propyl	**6e**	73
6	1	*i-*Propyl	**7b**	54
7	3	*i-*Propyl	**7d**	68

## Data Availability

Not applicable.

## Supplementary Materials

The following supporting information can be downloaded at: https://www.mdpi.com/article/10.3390/molecules27227687/s1, Figure S1: ^1^H NMR spectra (400 MHz, dmso) of 2-Bromo-*N*′-(2-bromoacetyl)acetohydrazide (**2a**); Figure S2: ^13^C NMR spectra (100 MHz, dmso) of 2-Bromo-*N*′-(2-bromoacetyl)acetohydrazide (**2a**); Figure S3: ^1^H NMR spectra (400 MHz, dmso) of 3-Bromo-*N*′-(3-bromopropanoyl)propanehydrazide (**2b**); Figure S4: ^13^C NMR spectra (100 MHz, dmso) of 3-Bromo-*N*′-(3-bromopropanoyl)propanehydrazide (**2b**); Figure S5: ^1^H NMR spectra (400 MHz, dmso) of 4-Bromo-*N*′-(4-bromobutanoyl)butanehydrazide (**2c**); Figure S6: ^13^C NMR spectra (100 MHz, dmso) of 4-Bromo-*N*′-(4-bromobutanoyl)butanehydrazide (**2c**); Figure S7: ^1^H NMR spectra (400 MHz, dmso) of 5-Bromo-*N*′-(5-bromopentanoyl)pentanehydrazide (**2d**); Figure S8: ^13^C NMR spectra (100 MHz, dmso) of 5-Bromo-*N*′-(5-bromopentanoyl)pentanehydrazide (**2d**); Figure S9: ^1^H NMR spectra (400 MHz, CDCl_3_) of 2,5-Bis(bromomethyl)-1,3,4-oxadiazole (**3a**); Figure S10: ^13^C NMR spectra (100 MHz, CDCl_3_) of 2,5-Bis(bromomethyl)-1,3,4-oxadiazole (**3a**); Figure S11: ^1^H NMR spectra (400 MHz, dmso) of 2,5-Bis(2-bromoethyl)-1,3,4-oxadiazole (**3b**); Figure S12: ^13^C NMR spectra (100 MHz, dmso) of 2,5-Bis(2-bromoethyl)-1,3,4-oxadiazole (**3b**); Figure S13: ^1^H NMR spectra (400 MHz, CDCl_3_) of 2,5-Bis(3-bromopropyl)-1,3,4-oxadiazole (**3c**); Figure S14: ^13^C NMR spectra (100 MHz, CDCl_3_) of 2,5-Bis(3-bromopropyl)-1,3,4-oxadiazole (**3c**); Figure S15: ^1^H NMR spectra (400 MHz, dmso) of 2,5-Bis(4-bromobutyl)-1,3,4-oxadiazole (**3d**); Figure S16: ^13^C NMR spectra (100 MHz, dmso) of 2,5-Bis(4-bromobutyl)-1,3,4-oxadiazole (**3d**); Figure S17: ^1^H NMR spectra (400 MHz, CDCl_3_) of Diethyl iminodiacetate (**5a**); Figure S18: ^13^C NMR spectra (100 MHz, CDCl_3_) of Diethyl iminodiacetate (**5a**); Figure S19: ^1^H NMR spectra (400 MHz, CDCl_3_) of Diisopropyl iminodiacetate (**5b**); Figure S20: ^13^C NMR spectra (100 MHz, CDCl_3_) of Diisopropyl iminodiacetate (**5b**); Figure S21: ^1^H NMR spectra (400 MHz, CDCl_3_) of Tetraethyl 2,2′,2″,2‴-(((1,3,4-oxadiazole-2,5-diyl)bis(methylene))bis(azanetriyl))tetraacetate (**6a**); Figure S22: ^13^C NMR spectra (100 MHz, CDCl_3_) of Tetraethyl 2,2′,2″,2‴-(((1,3,4-oxadiazole-2,5-diyl)bis(methylene))bis(azanetriyl))tetraacetate (**6a**); Figure S23: ^1^H NMR spectra (400 MHz, CDCl_3_) of Tetraisopropyl 2,2′,2″,2‴-(((1,3,4-oxadiazole=2,5-diyl)bis(methylene))bis(azanetriyl))tetraacetate (**6b**); Figure S24: ^13^C NMR spectra (100 MHz, CDCl_3_) of Tetraisopropyl 2,2′,2″,2‴-(((1,3,4-oxadiazole=2,5-diyl)bis(methylene))bis(azanetriyl))tetraacetate (**6b**); Figure S25: ^1^H NMR spectra (400 MHz, CDCl_3_) of Tetraisopropyl 2,2′,2″,2‴-(((1,3,4-oxadiazole-2,5-diyl)bis(ethane-2,1-diyl))bis(azanetriyl))tetraacete (**6c**); Figure S26: ^13^C NMR spectra (100 MHz, CDCl_3_) of Tetraisopropyl 2,2′,2″,2‴-(((1,3,4-oxadiazole-2,5-diyl)bis(ethane-2,1-diyl))bis(azanetriyl))tetraacete (**6c**); Figure S27: ^1^H NMR spectra (400 MHz, CDCl_3_) of Tetraisopropyl 2,2′,2″,2‴-(((1,3,4-oxadiazole-2,5-diyl)bis(propane-3,1-diyl))bis(azanetriyl))tetraacetate (**6d**); Figure S28: ^13^C NMR spectra (100 MHz, CDCl_3_) of Tetraisopropyl 2,2′,2″,2‴-(((1,3,4-oxadiazole-2,5-diyl)bis(propane-3,1-diyl))bis(azanetriyl))tetraacetate (**6d**); Figure S29: ^1^H NMR spectra (400 MHz, CDCl_3_) of Tetraisopropyl 2,2′,2″,2‴-(((1,3,4-oxadiazole-2,5-diyl)bis(butane-4,1-diyl))bis(azanetriyl))tetraacetate (**6e**); Figure S30: ^13^C NMR spectra (100 MHz, CDCl_3_) of Tetraisopropyl 2,2′,2″,2‴-(((1,3,4-oxadiazole-2,5-diyl)bis(butane-4,1-diyl))bis(azanetriyl))tetraacetate (**6e**); Figure S31: ^1^H NMR spectra (400 MHz, dmso) of 2,2′,2″,2‴-(((1,3,4-oxadiazole-2,5-diyl)bis(methylene))bis(azanetriyl))tetraacetic acid (**7b**); Figure S32: ^13^C NMR spectra (400 MHz, dmso) of 2,2′,2″,2‴-(((1,3,4-oxadiazole-2,5-diyl)bis(methylene))bis(azanetriyl))tetraacetic acid (**7b**); Figure S33: ^1^H NMR spectra (100 MHz, dmso) of 2,2′,2″,2‴-(((1,3,4-oxadiazole-2,5-diyl)bis(propane-3,1-diyl))bis(azanetriyl))tetraacetic acid (**7d**); Figure S34: ^13^C NMR spectra (400 MHz, dmso) of 2,2′,2″,2‴-(((1,3,4-oxadiazole-2,5-diyl)bis(propane-3,1-diyl))bis(azanetriyl))tetraacetic acid (**7d**).
