# Supplementary material for: Synthesis of 2,5-Dialkyl-1,3,4-oxadiazoles Bearing Carboxymethylamino Groups
Source: Molecules. 2022 Nov 9;27(22):7687. doi: 10.3390/molecules27227687 (PMC9696334; doi:10.3390/molecules27227687)
Supplement: Supplementary file 1 [file molecules-27-07687-s001.zip › molecules-2009956-supplementary.pdf]

Supplementary Materials

Synthesis of 2,5-dialkyl-1,3,4-oxadiazoles bearing carboxymethylamino groups

Marcin Łuczyński [1], Kornelia Kubiesa [1], and Agnieszka Kudelko [1,*]

[1] Department of Chemical Organic Technology and Petrochemistry, The Silesian University of Technology, Krzywoustego 4, PL-44100 Gliwice, Poland

1. ^{1}H and ^{13}C NMR spectra

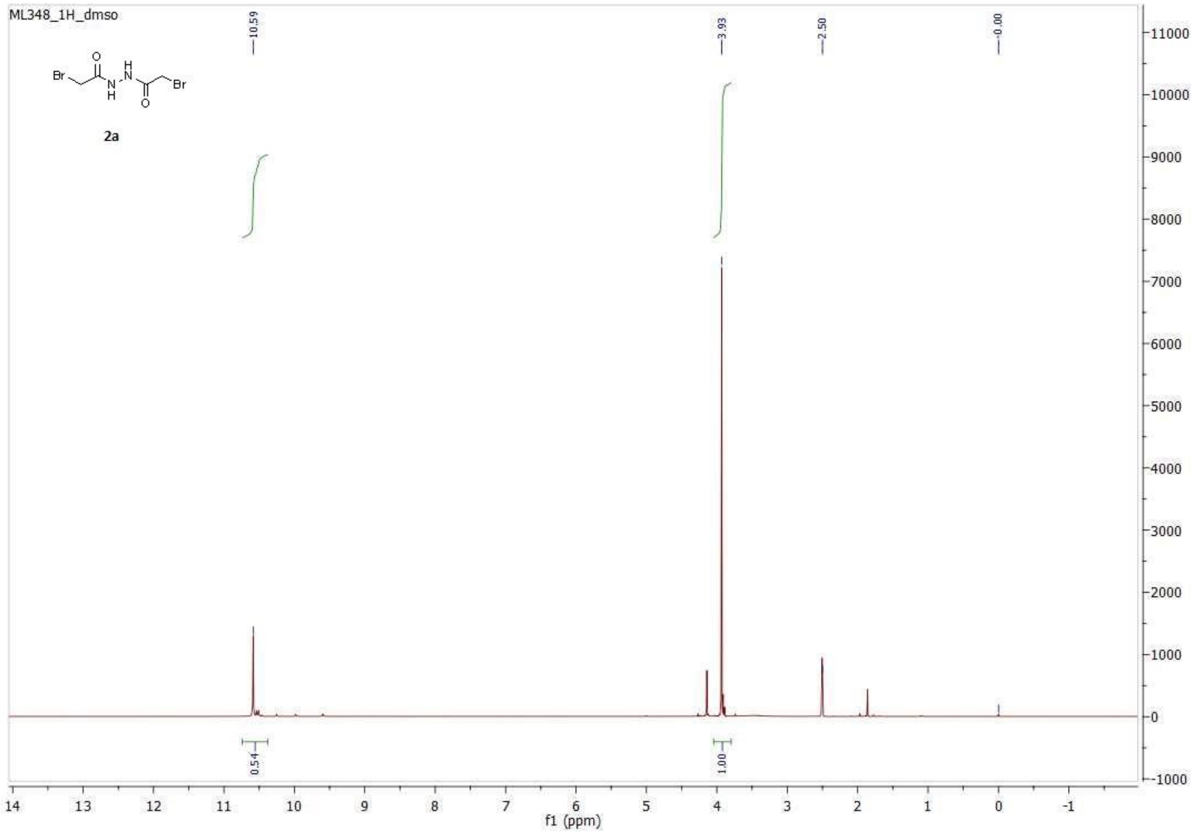

Figure S1. ^{1}H NMR spectra (400 MHz, dmso) of 2-Bromo-*N*'-(2-bromoacetyl)acetohydrazide (**2a**)

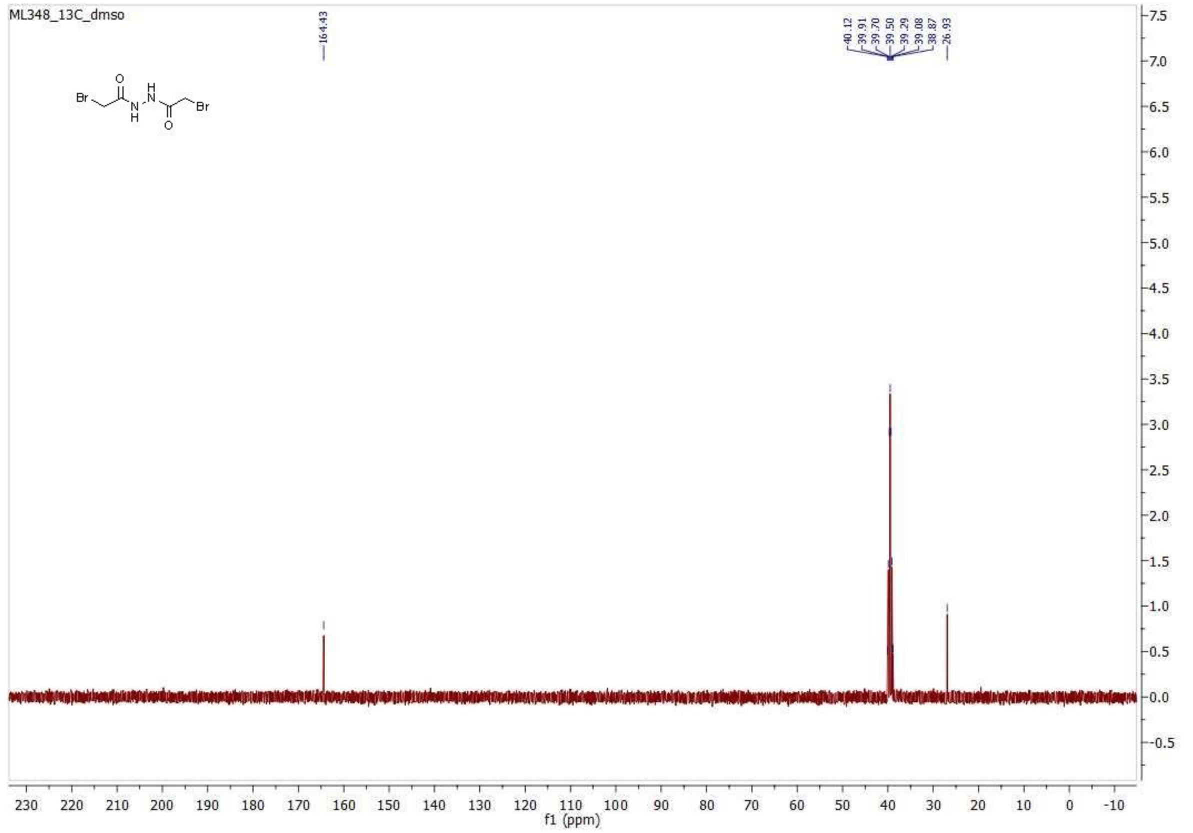

Figure S2. ^{13}C NMR spectra (100 MHz, dmso) of 2-Bromo-*N*'-(2-bromoacetyl)acetohydrazide (**2a**)

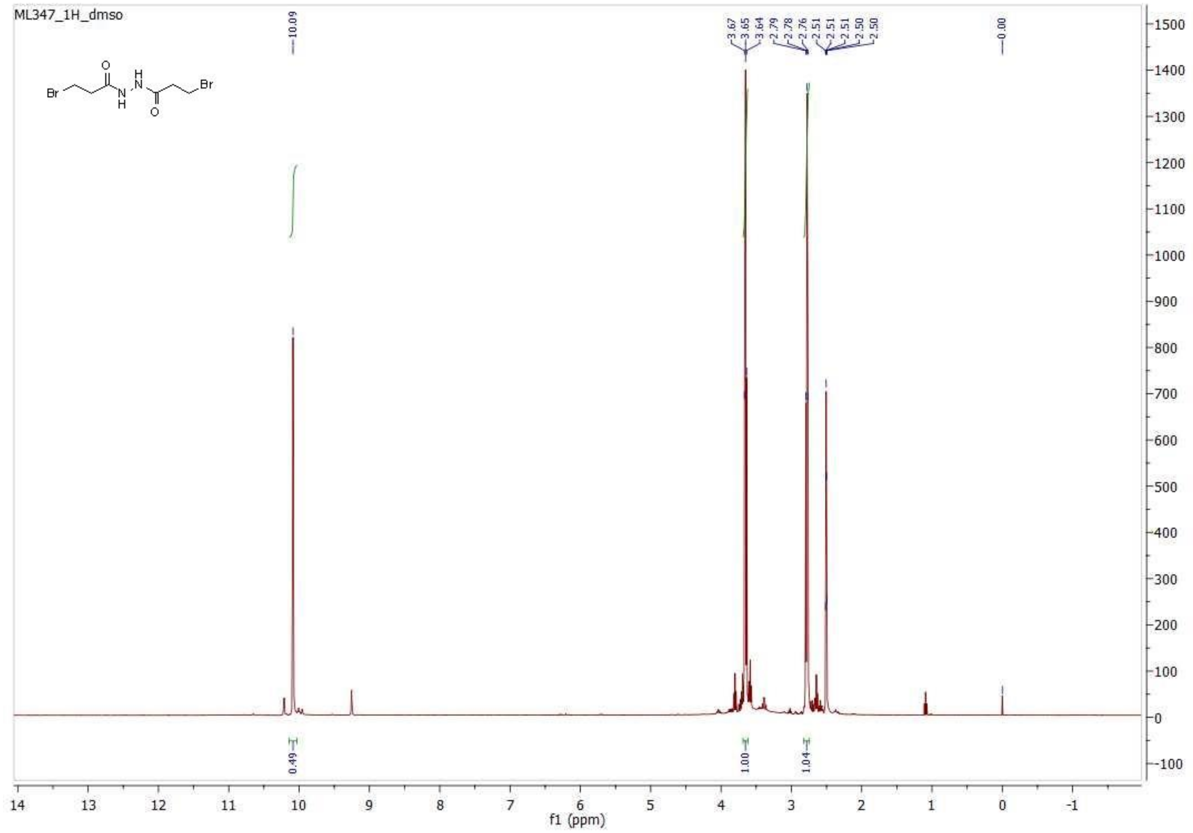

Figure S3. ^{1}H NMR spectra (400 MHz, dmso) of 3-Bromo-*N'*-(3-bromopropanoyl)propanehydrazide (**2b**)

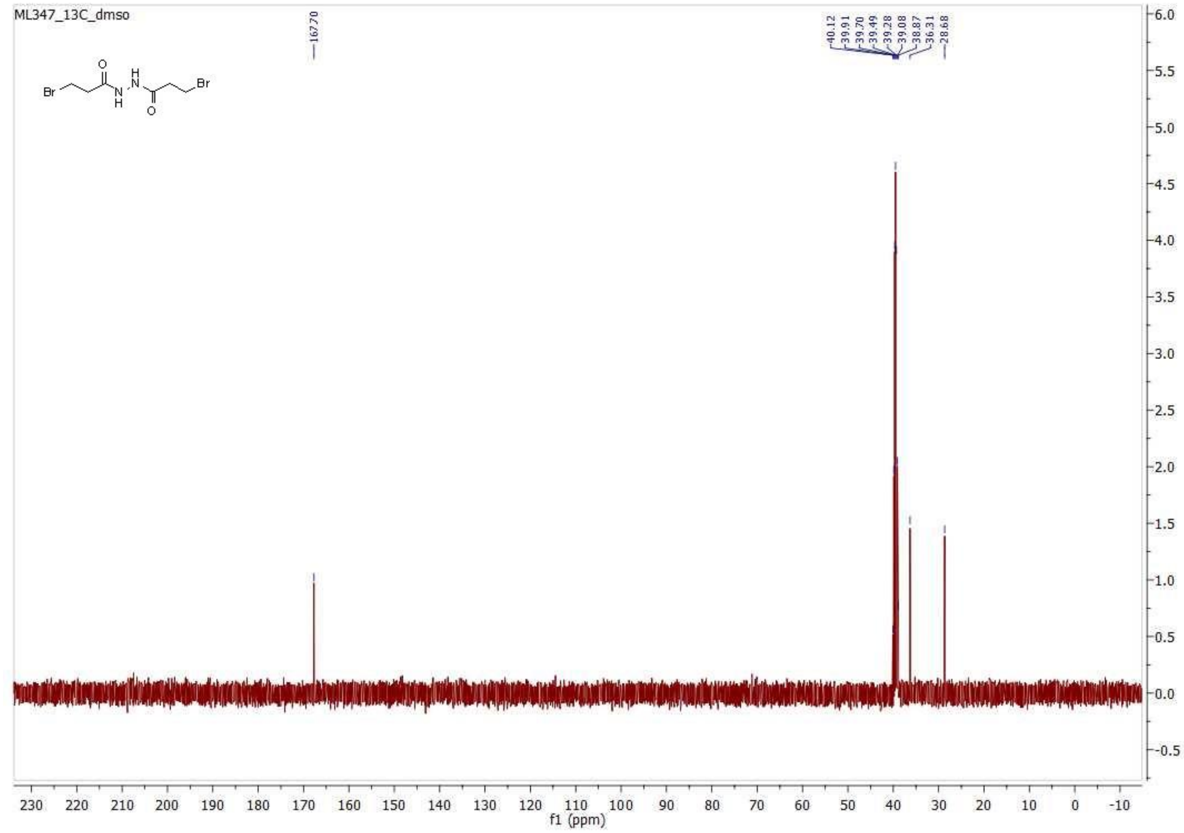

Figure S4. ^{13}C NMR spectra (100 MHz, dmso) of 3-Bromo-*N'*-(3-bromopropanoyl)propanehydrazide (**2b**)

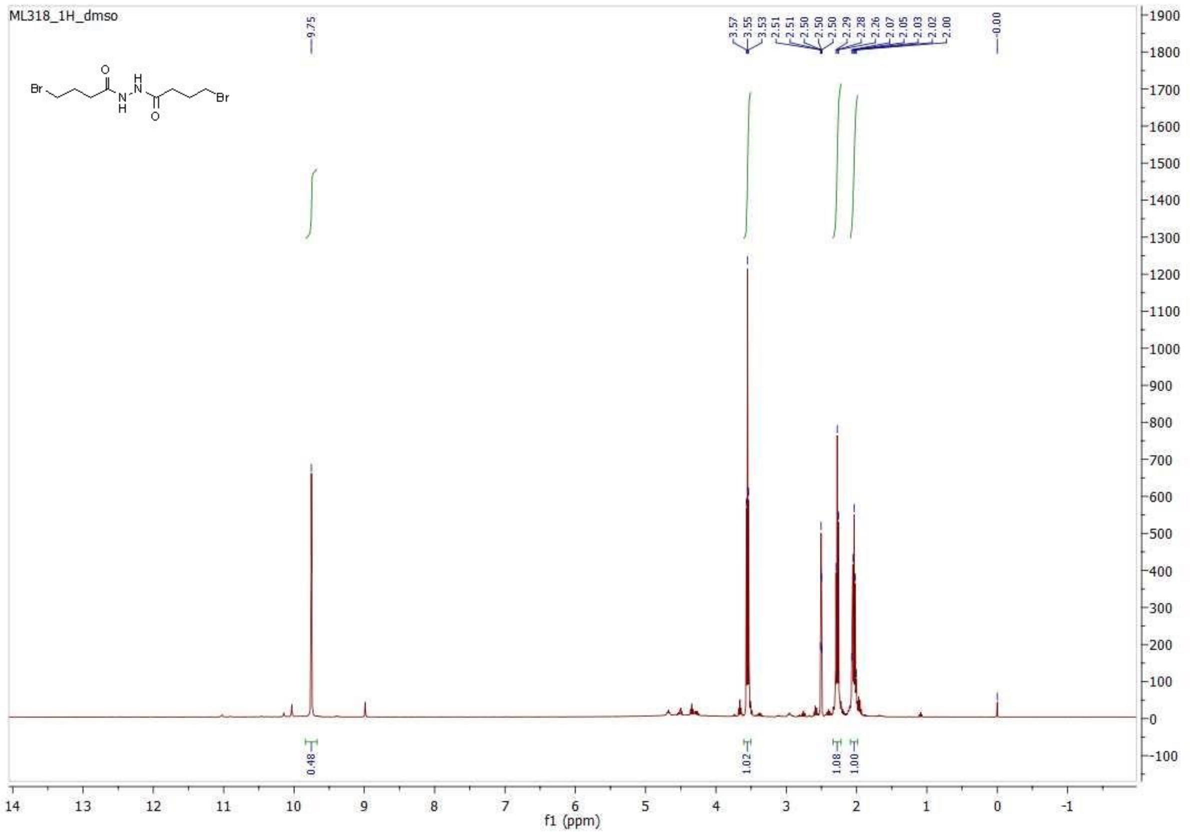

Figure S5. ^{1}H NMR spectra (400 MHz, dmso) of 4-Bromo-*N'*-(4-bromobutanoyl)butanehydrazide (**2c**)

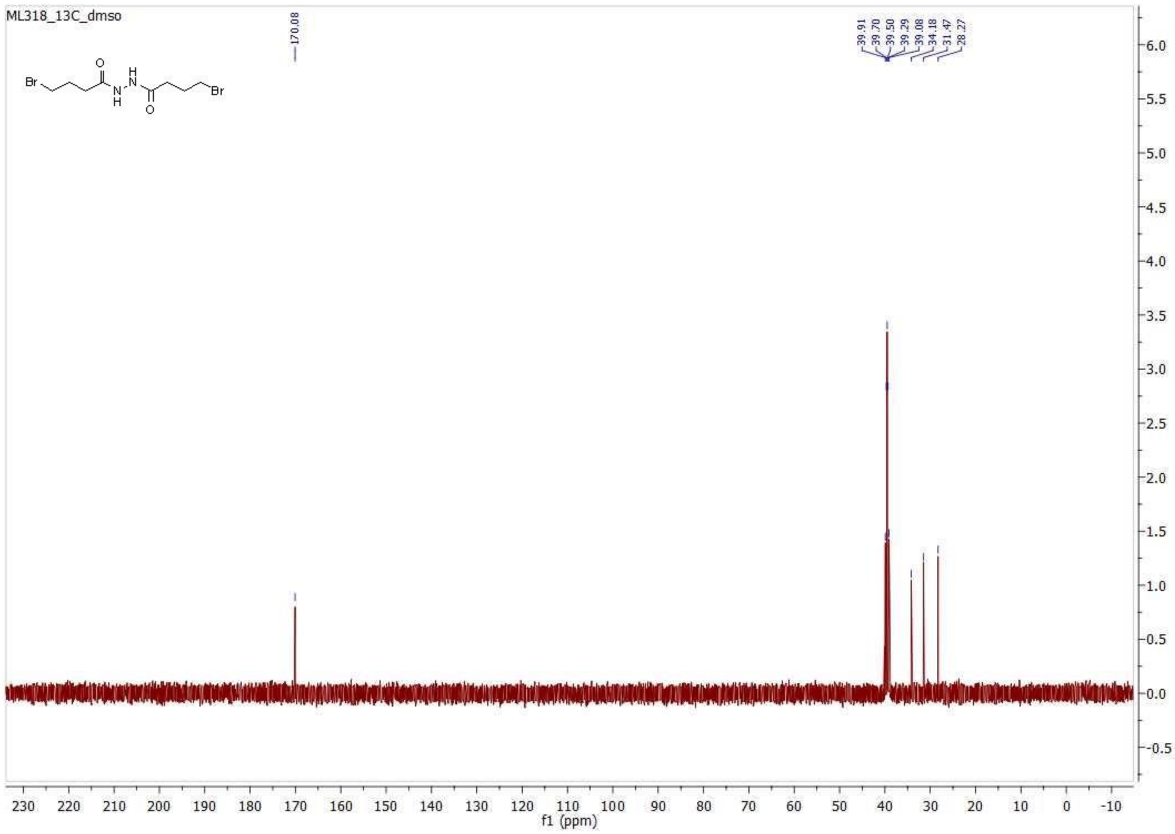

Figure S6. ^{13}C NMR spectra (100 MHz, dmso) of 4-Bromo-*N'*-(4-bromobutanoyl)butanehydrazide (**2c**)

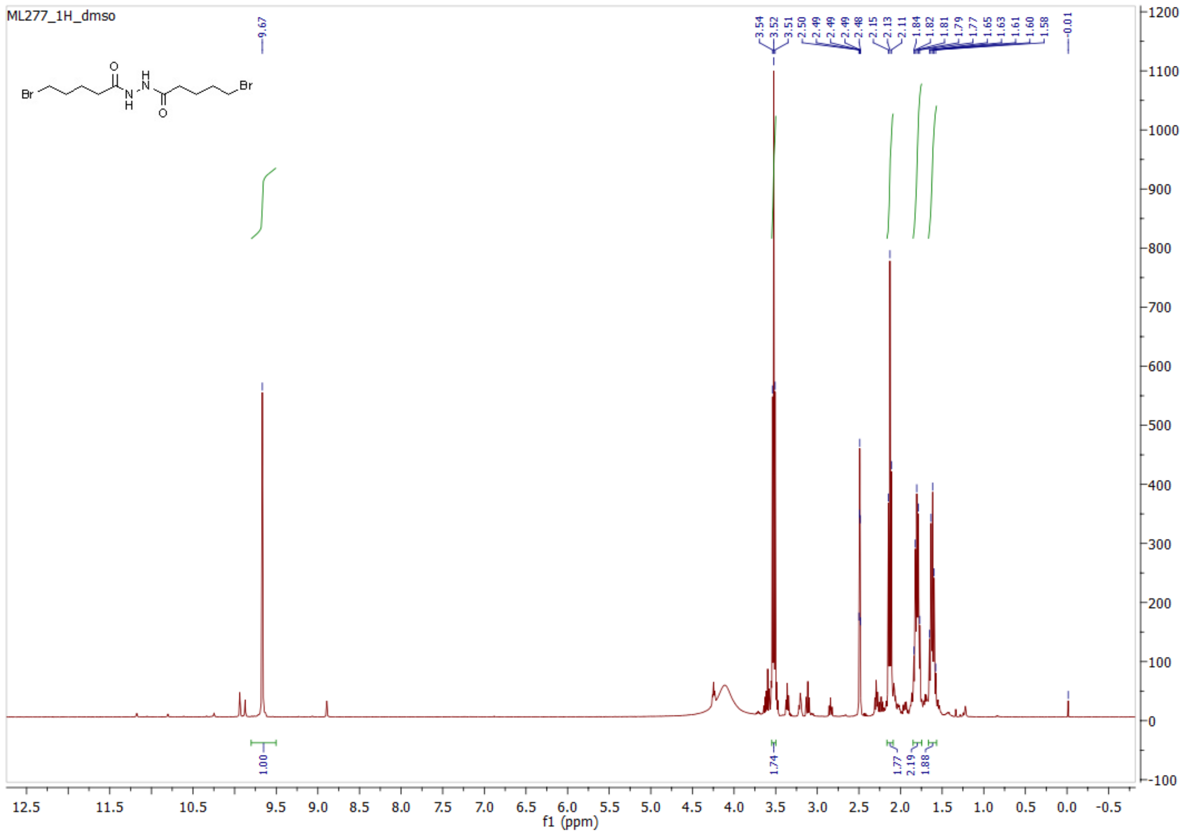

Figure S7. ^{1}H NMR spectra (400 MHz, dmso) of 5-Bromo-*N'*-(5-bromopentanoyl)pentanehydrazide (**2d**)

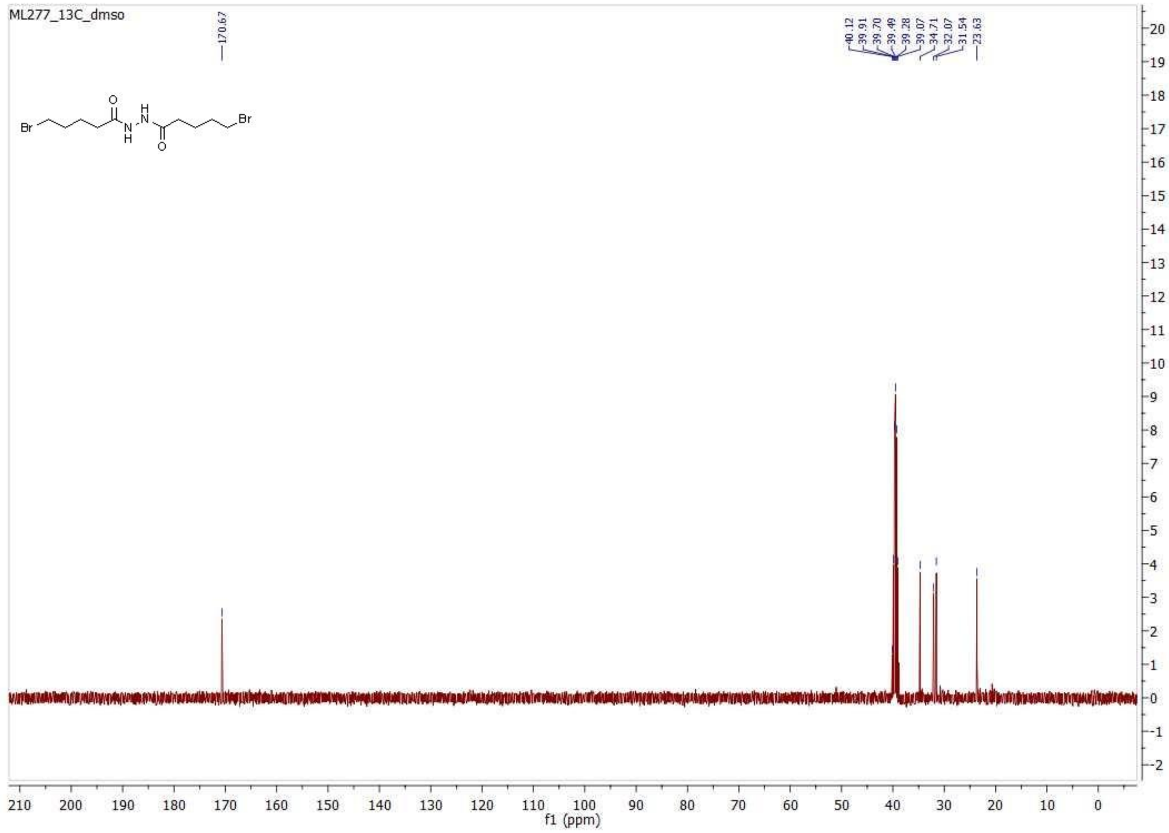

Figure S8. ^{13}C NMR spectra (100 MHz, dmso) of 5-Bromo-*N′*-(5-bromopentanoyl)pentanehydrazide (**2d**)

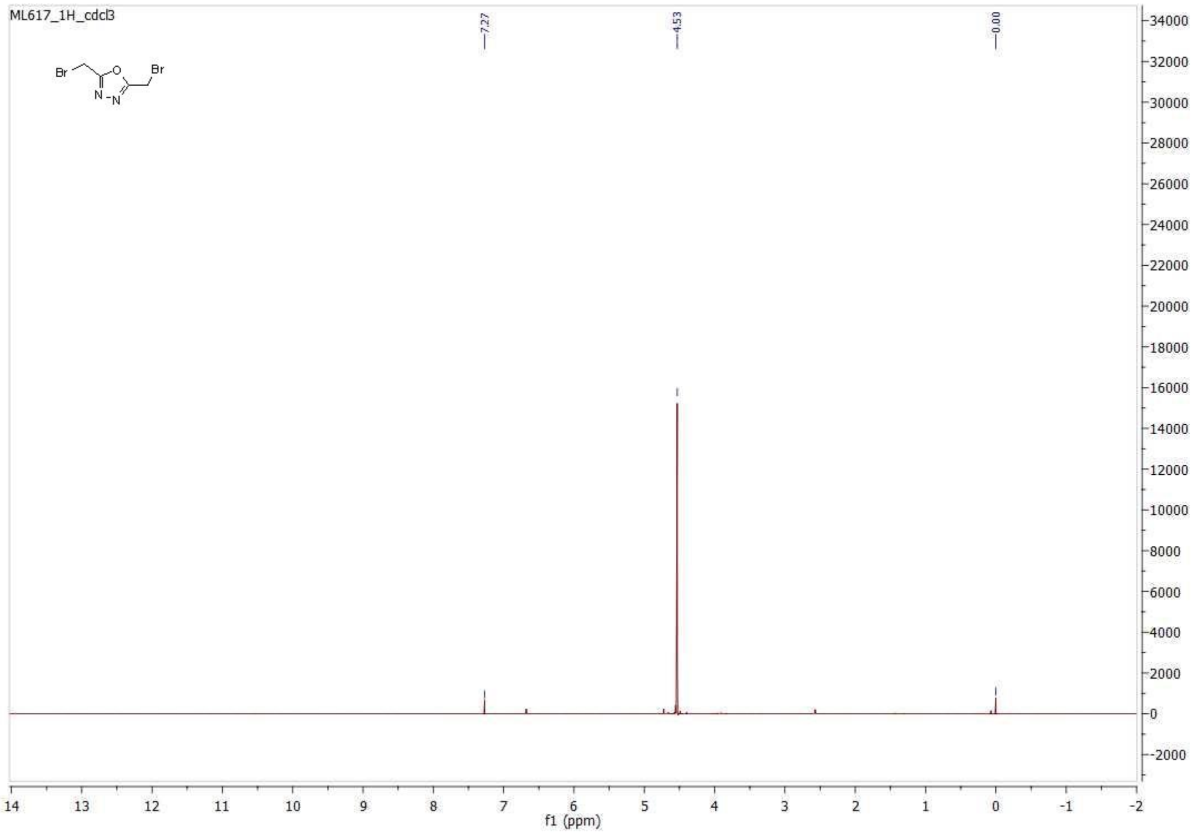

Figure S9. ^{1}H NMR spectra (400 MHz, $CDCl_3$) of 2,5-Bis(bromomethyl)-1,3,4-oxadiazole (**3a**)

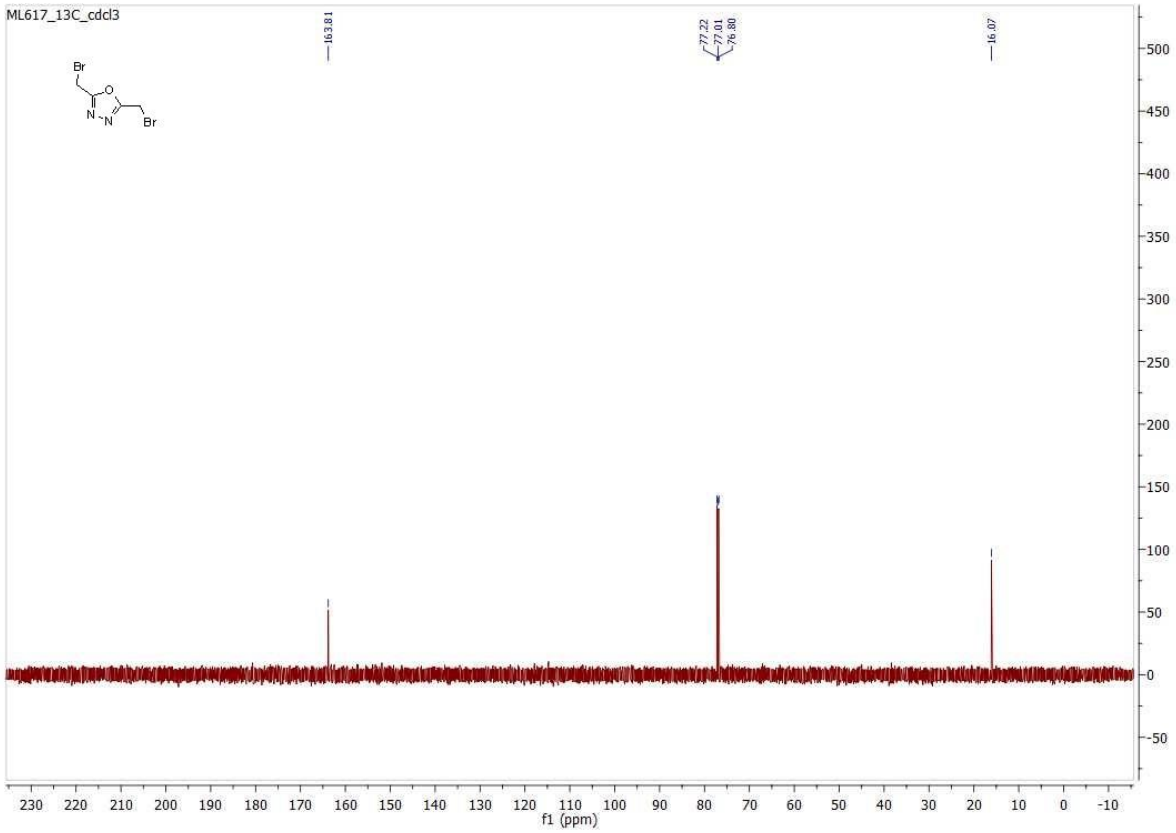

Figure S10. ^{13}C NMR spectra (100 MHz, $CDCl_3$) of 2,5-Bis(bromomethyl)-1,3,4-oxadiazole (**3a**)

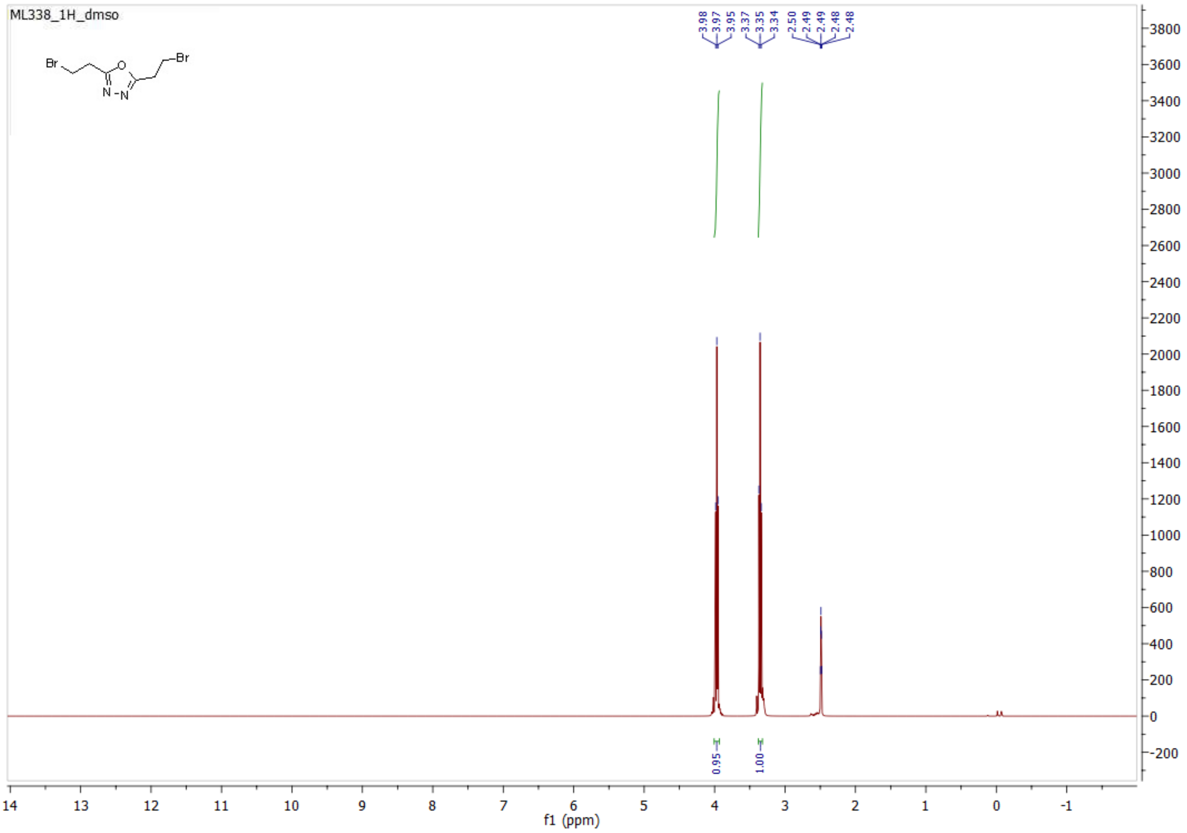

Figure S11. ^{1}H NMR spectra (400 MHz, dmso) of 2,5-Bis(2-bromoethyl)-1,3,4-oxadiazole (**3b**)

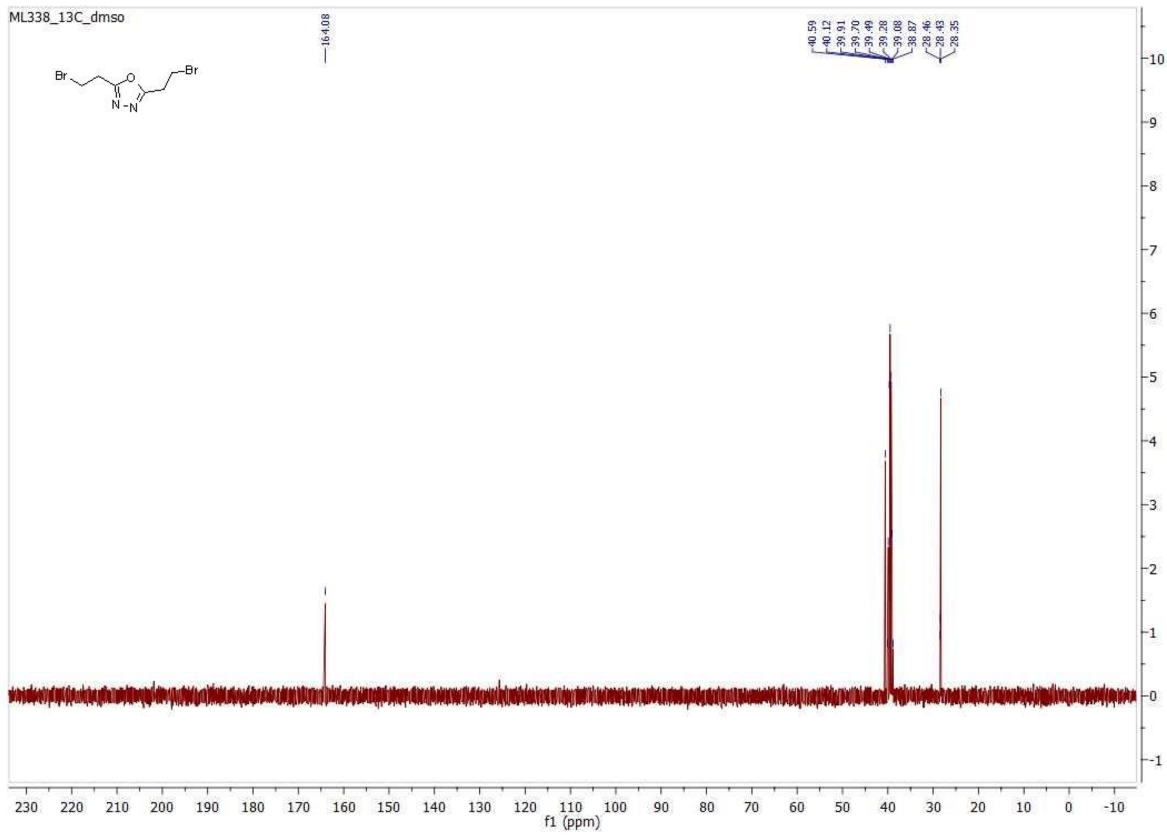

Figure S12. ^{13}C NMR spectra (100 MHz, dmso) of 2,5-Bis(2-bromoethyl)-1,3,4-oxadiazole (**3b**)

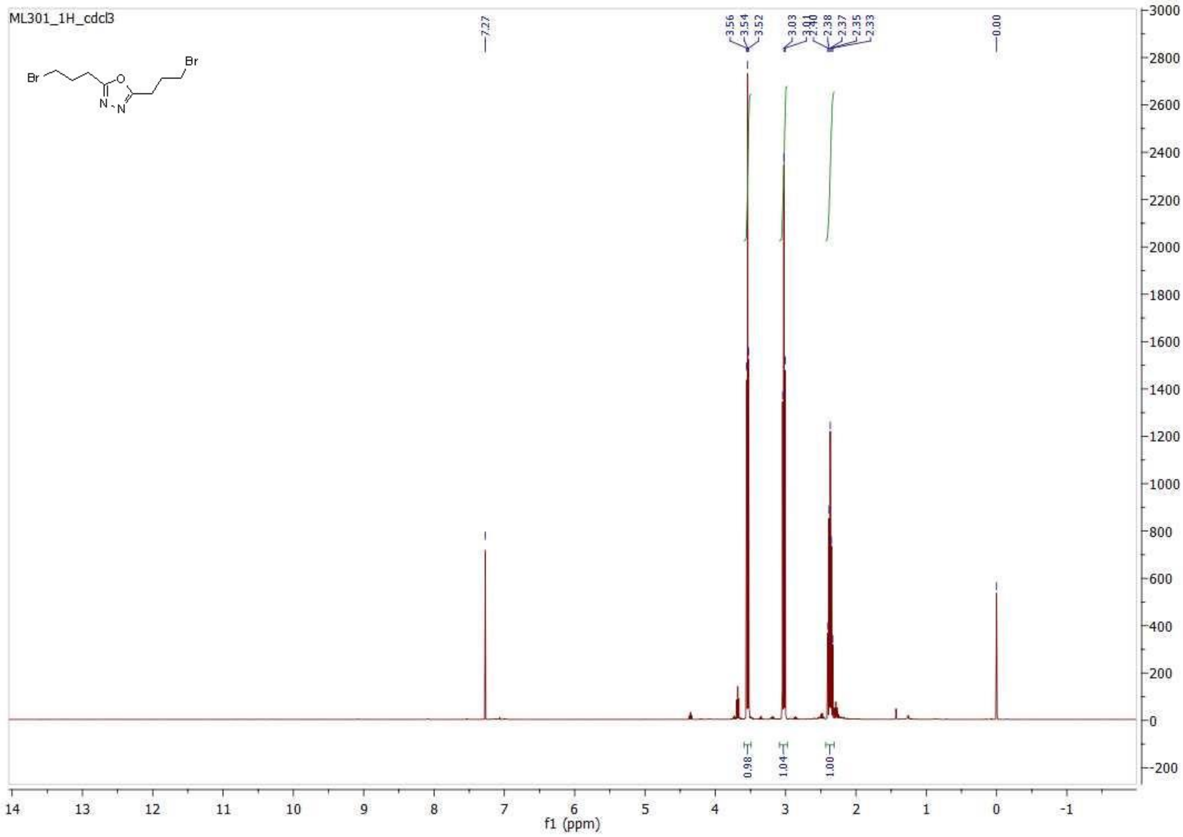

Figure S13. ^{1}H NMR spectra (400 MHz, $CDCl_3$) of 2,5-Bis(3-bromopropyl)-1,3,4-oxadiazole (**3c**)

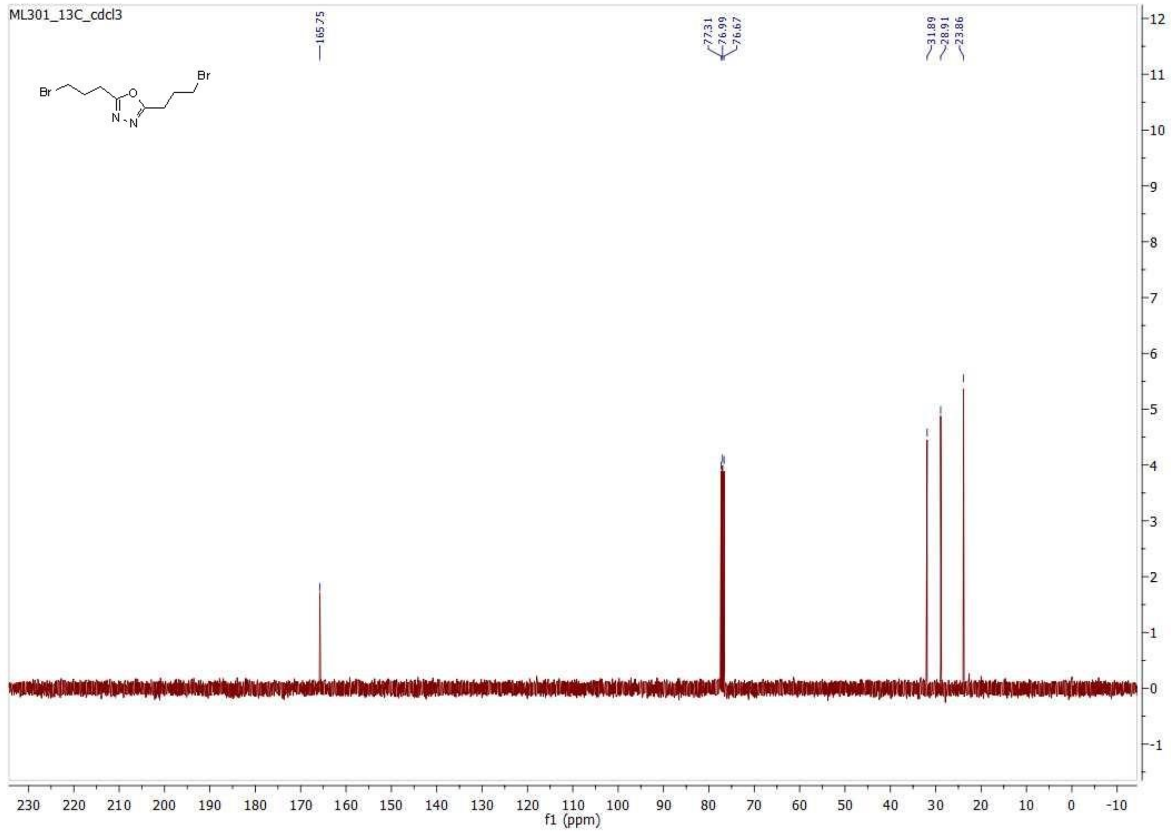

Figure S14. ^{13}C NMR spectra (100 MHz, $CDCl_3$) of 2,5-Bis(3-bromopropyl)-1,3,4-oxadiazole (**3c**)

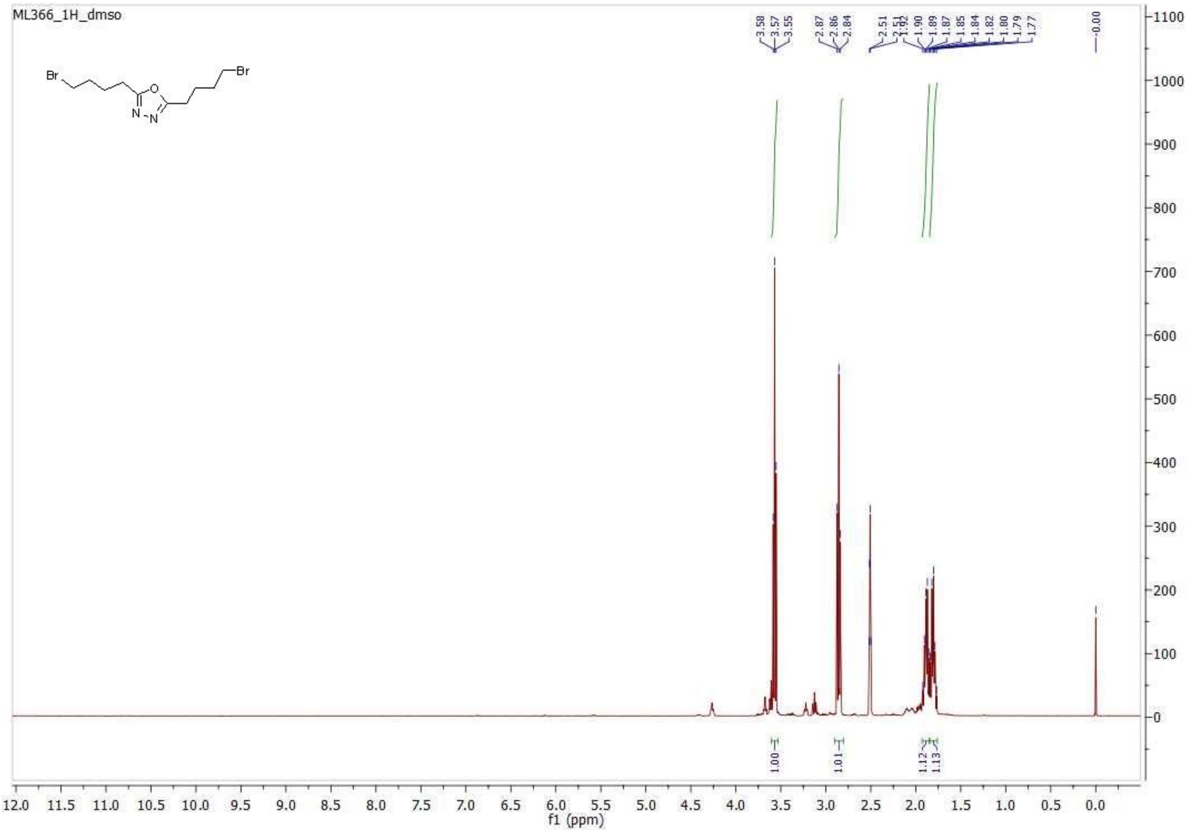

Figure S15. ^{1}H NMR spectra (400 MHz,dmso) of 2,5-Bis(4-bromobutyl)-1,3,4-oxadiazole (**3d**)

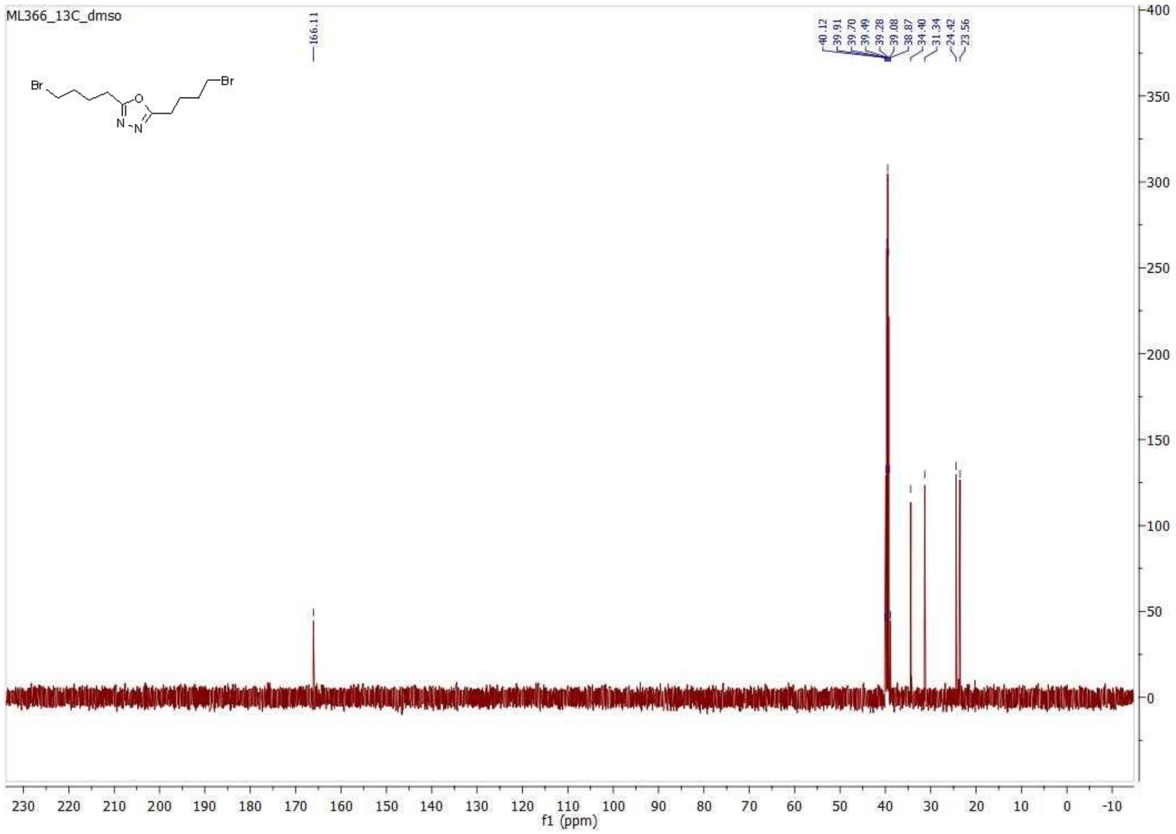

Figure S16. ^{13}C NMR spectra (100 MHz,dmso) of 2,5-Bis(4-bromobutyl)-1,3,4-oxadiazole (**3d**)

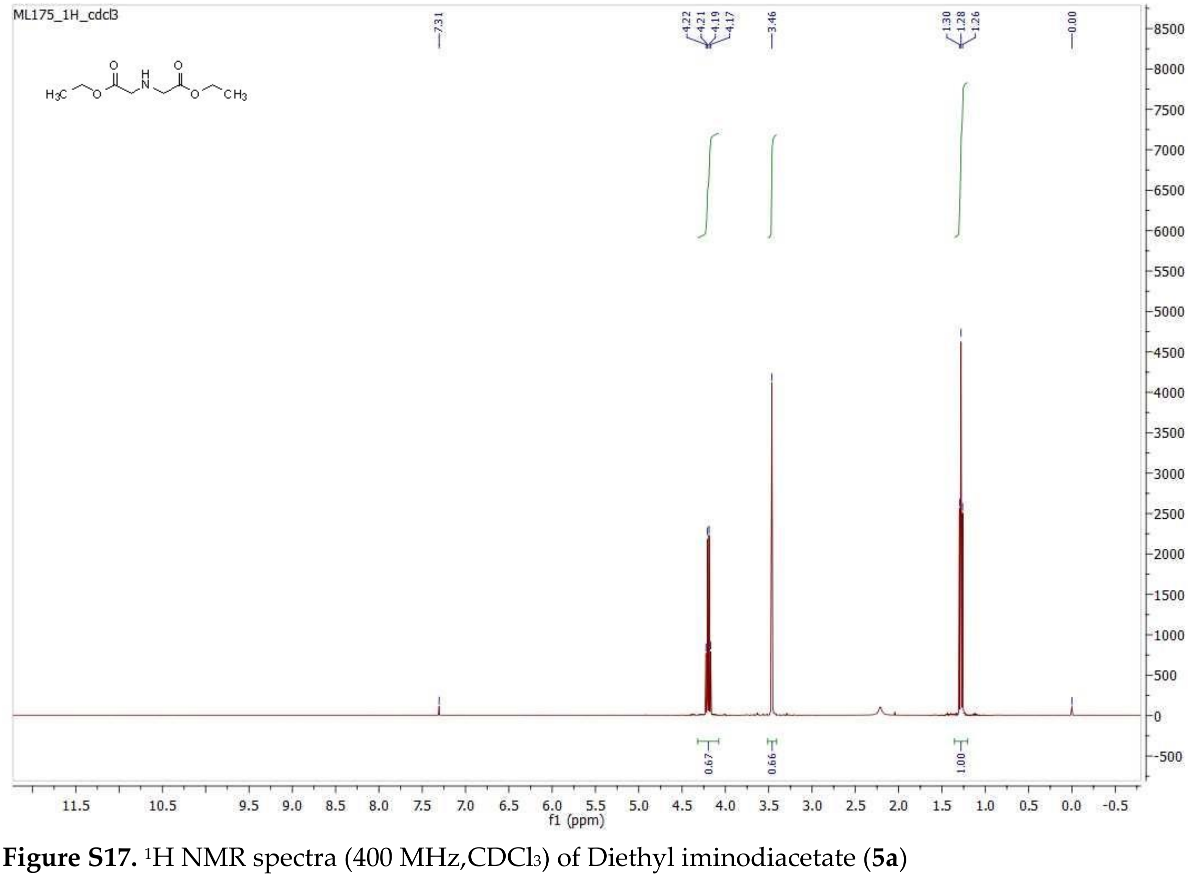

Figure S17. ^{1}H NMR spectra (400 MHz,$CDCl_3$) of Diethyl iminodiacetate (**5a**)

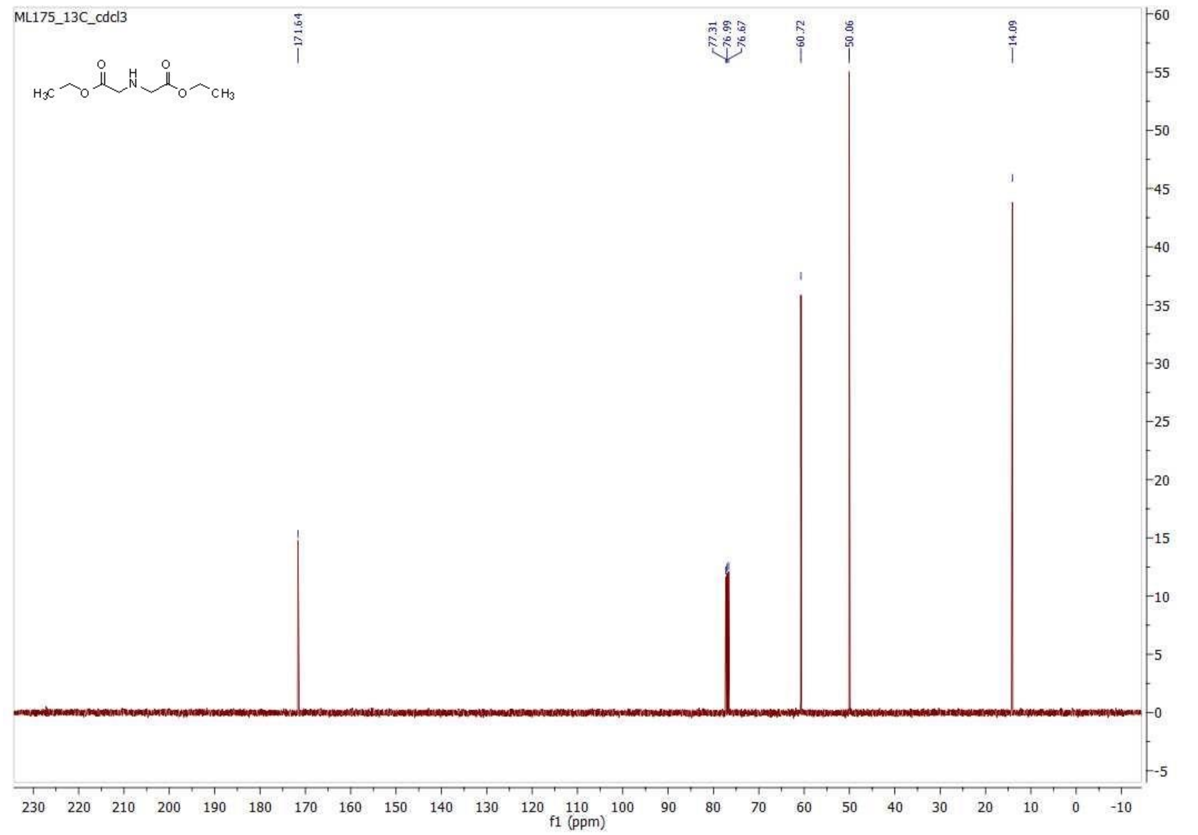

Figure S18. ^{13}C NMR spectra (100 MHz,$CDCl_3$) of Diethyl iminodiacetate (**5a**)

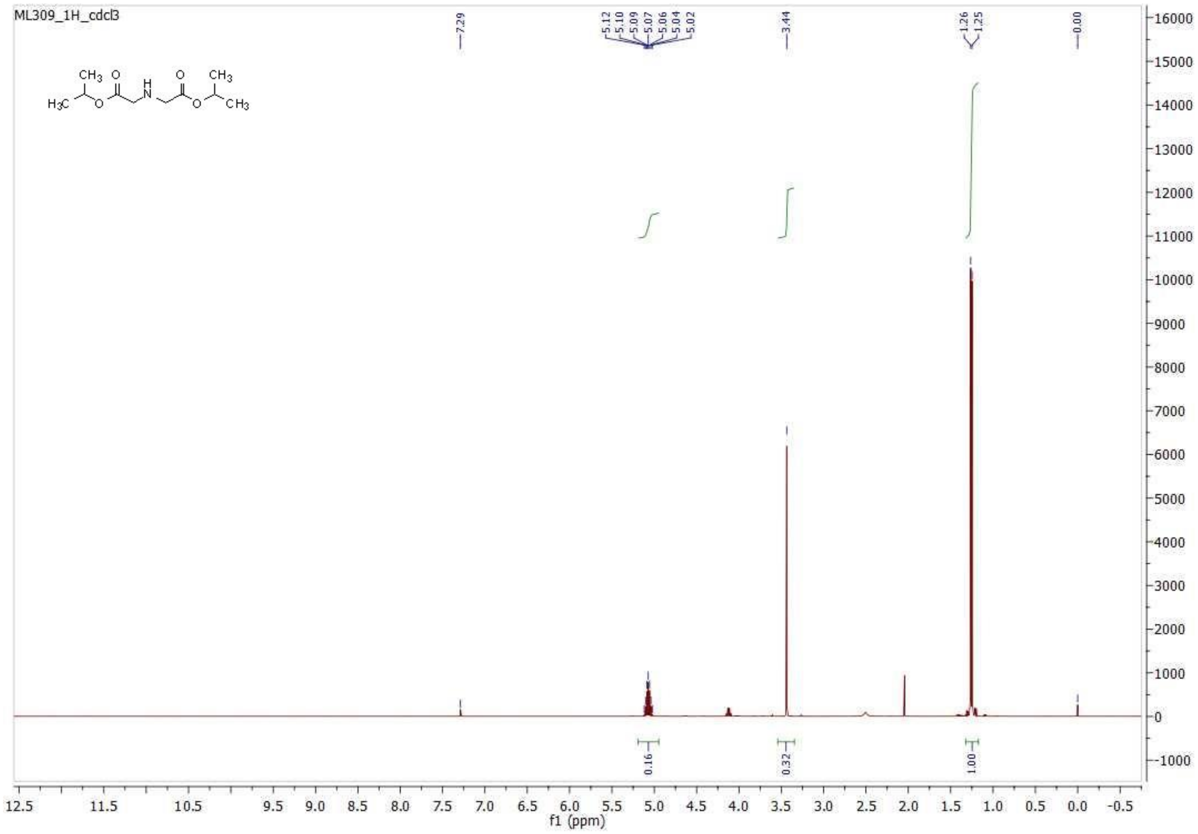

Figure S19. ^{1}H NMR spectra (400 MHz,$CDCl_3$) of Diisopropyl iminodiacetate (**5b**)

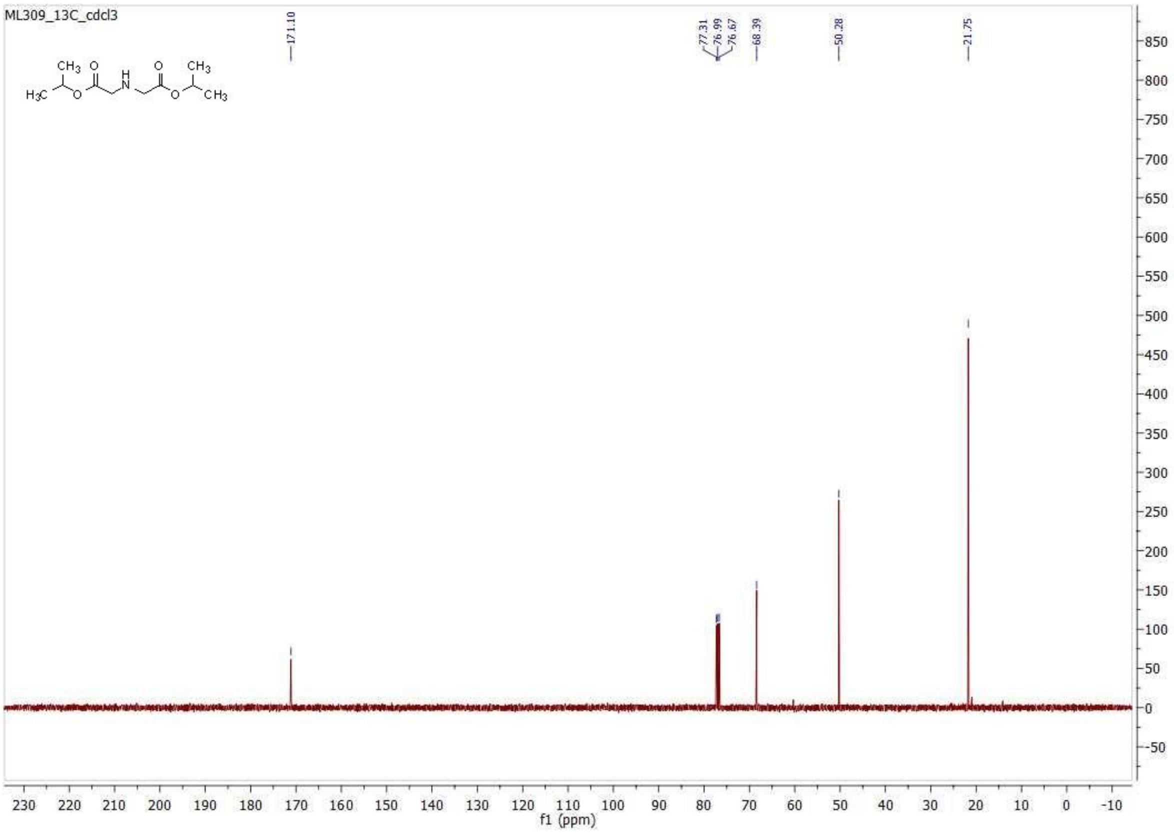

Figure S20. ^{13}C NMR spectra (100 MHz,$CDCl_3$) of Diisopropyl iminodiacetate (**5b**)

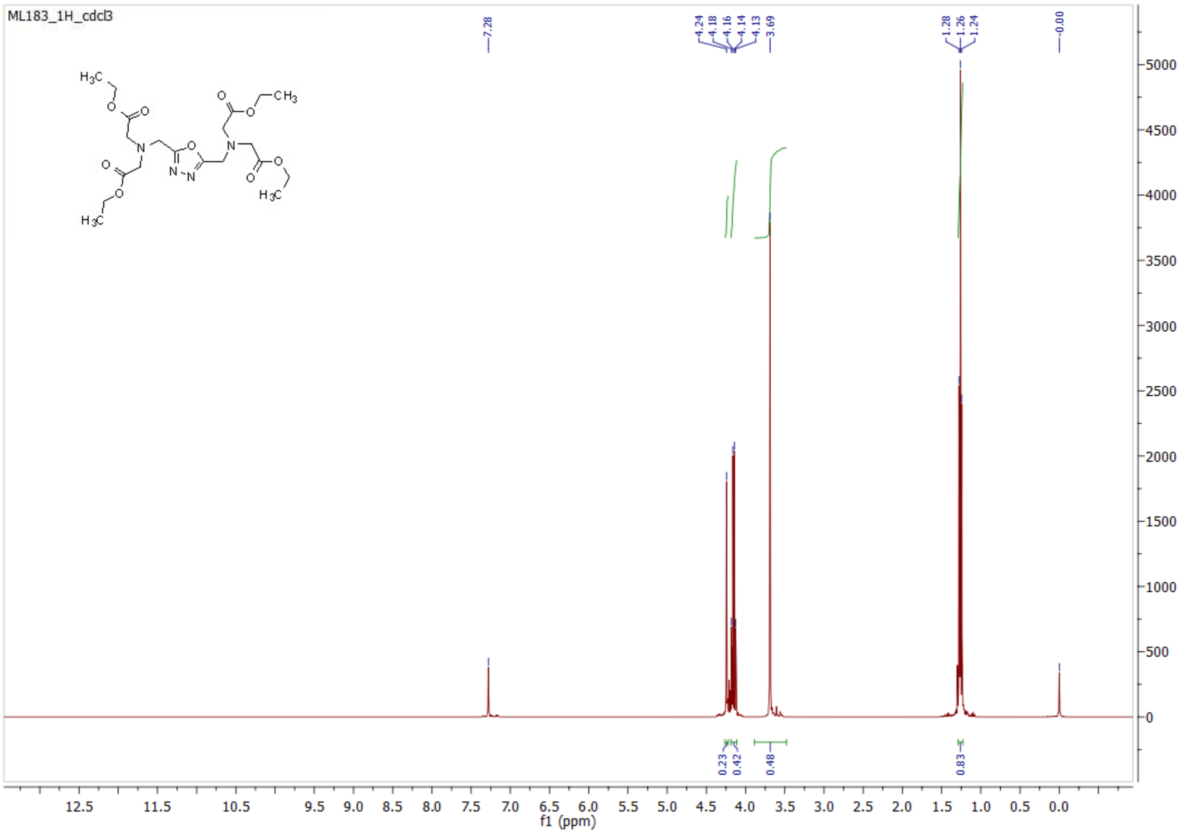

Figure S21. ^{1}H NMR spectra (400 MHz,$CDCl_3$) of Tetraethyl 2,2′,2′′,2′′′-(((1,3,4-oxadiazole-2,5-diyl)bis(methylene))bis(azanetriyl))tetraacetate (**6a**)

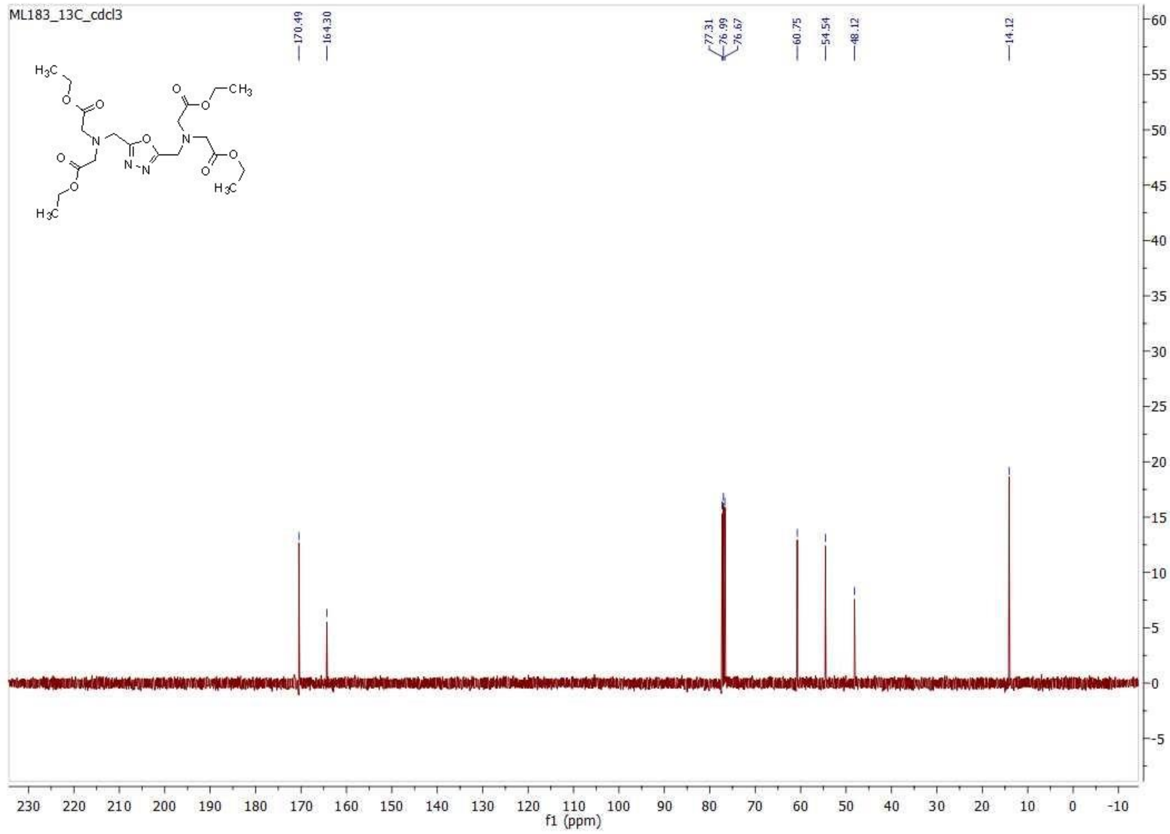

Figure S22. ^{13}C NMR spectra (100 MHz,$CDCl_3$) of Tetraethyl 2,2′,2′′,2′′′-(((1,3,4-oxadiazole-2,5-diyl)bis(methylene))bis(azanetriyl))tetraacetate (**6a**)

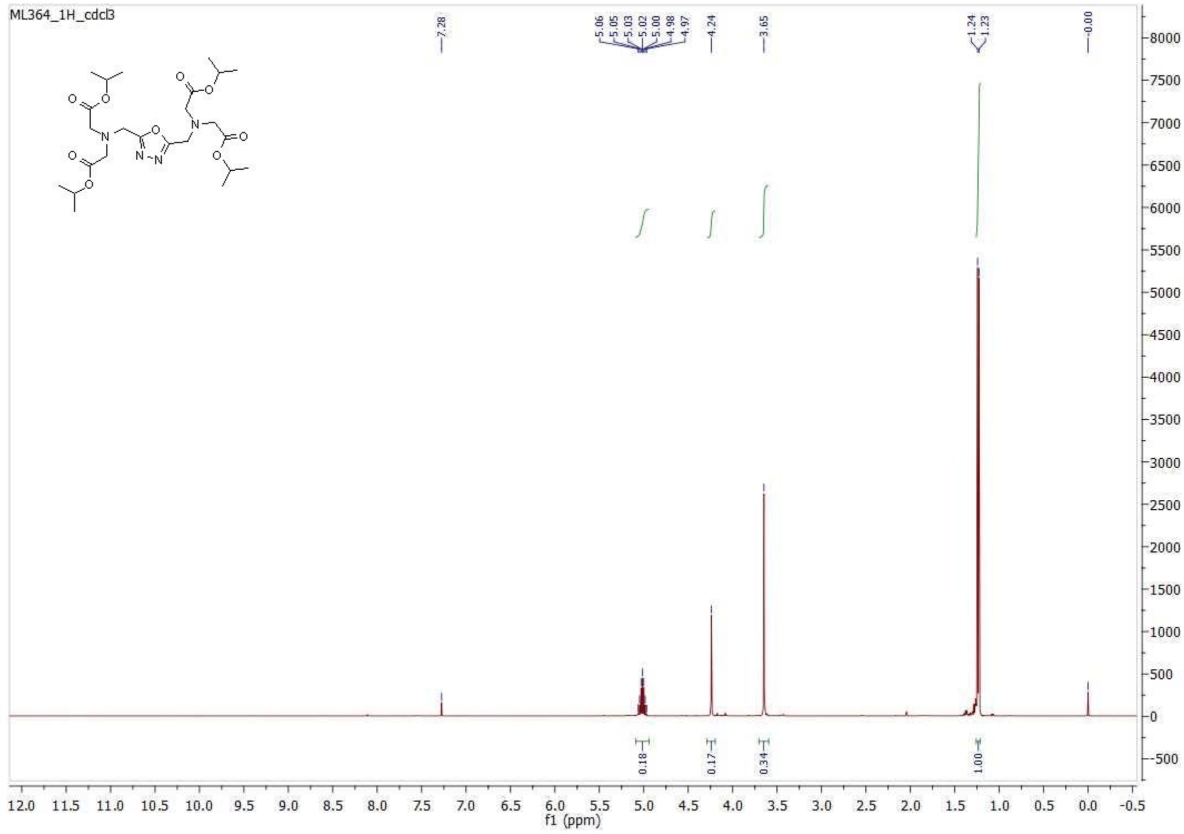

Figure S23. ^{1}H NMR spectra (400 MHz,$CDCl_3$) of Tetraisopropyl 2,2′,2′′,2′′′-(((1,3,4-oxadiazole=2,5-diyl)bis(methylene))bis(azanetriyl))tetraacetate (**6b**)

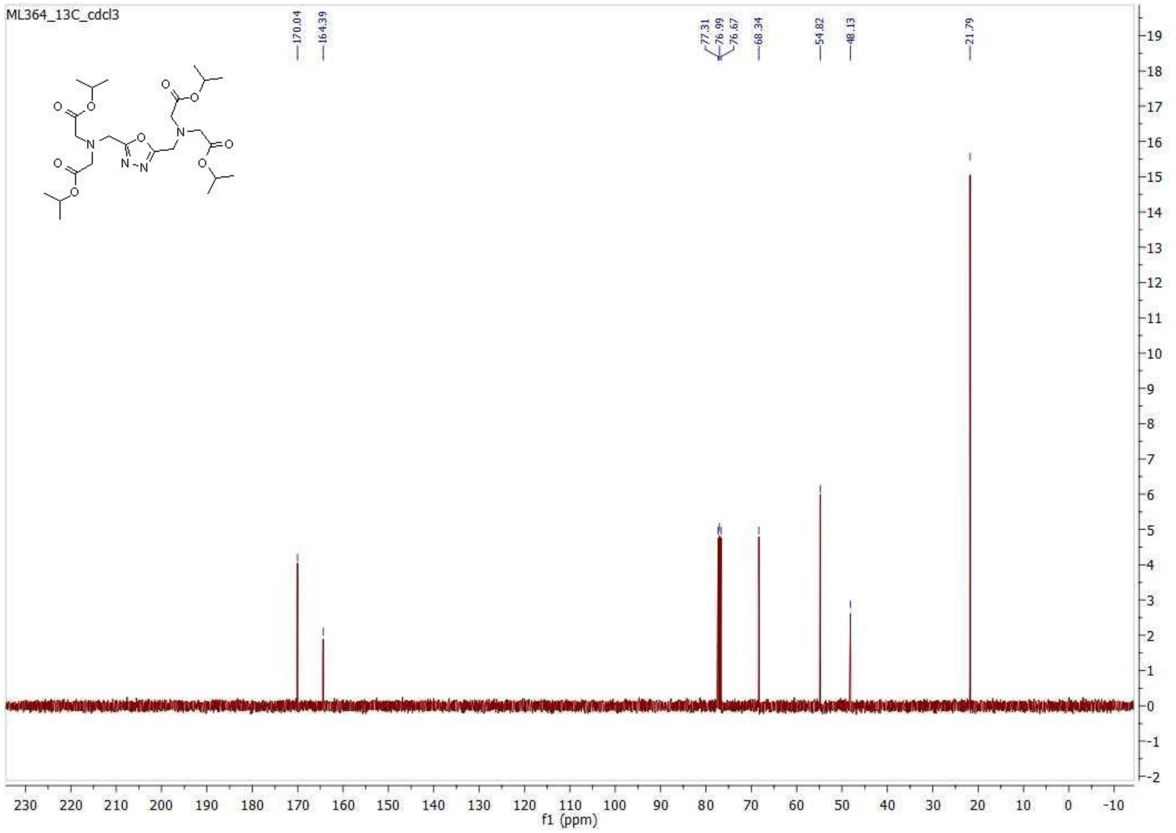

Figure S24. ^{13}C NMR spectra (100 MHz,$CDCl_3$) of Tetraisopropyl 2,2′,2′′,2′′′-(((1,3,4-oxadiazole=2,5-diyl)bis(methylene))bis(azanetriyl))tetraacetate (**6b**)

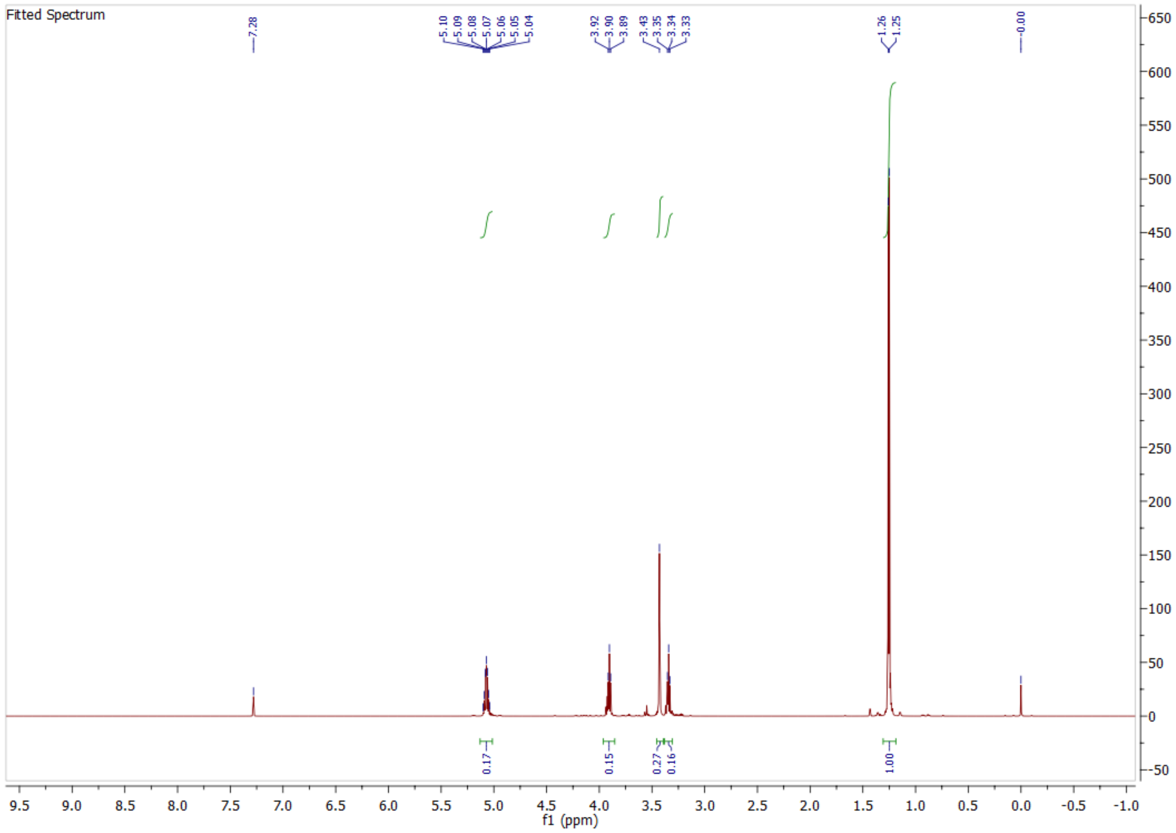

Figure S25. ^{1}H NMR spectra (400 MHz,$CDCl_3$) of Tetraisopropyl 2,2′,2′′,2′′′-(((1,3,4-oxadiazole-2,5-diyl)bis(ethane-2,1-diyl))bis(azanetriyl))tetraacete (**6c**)

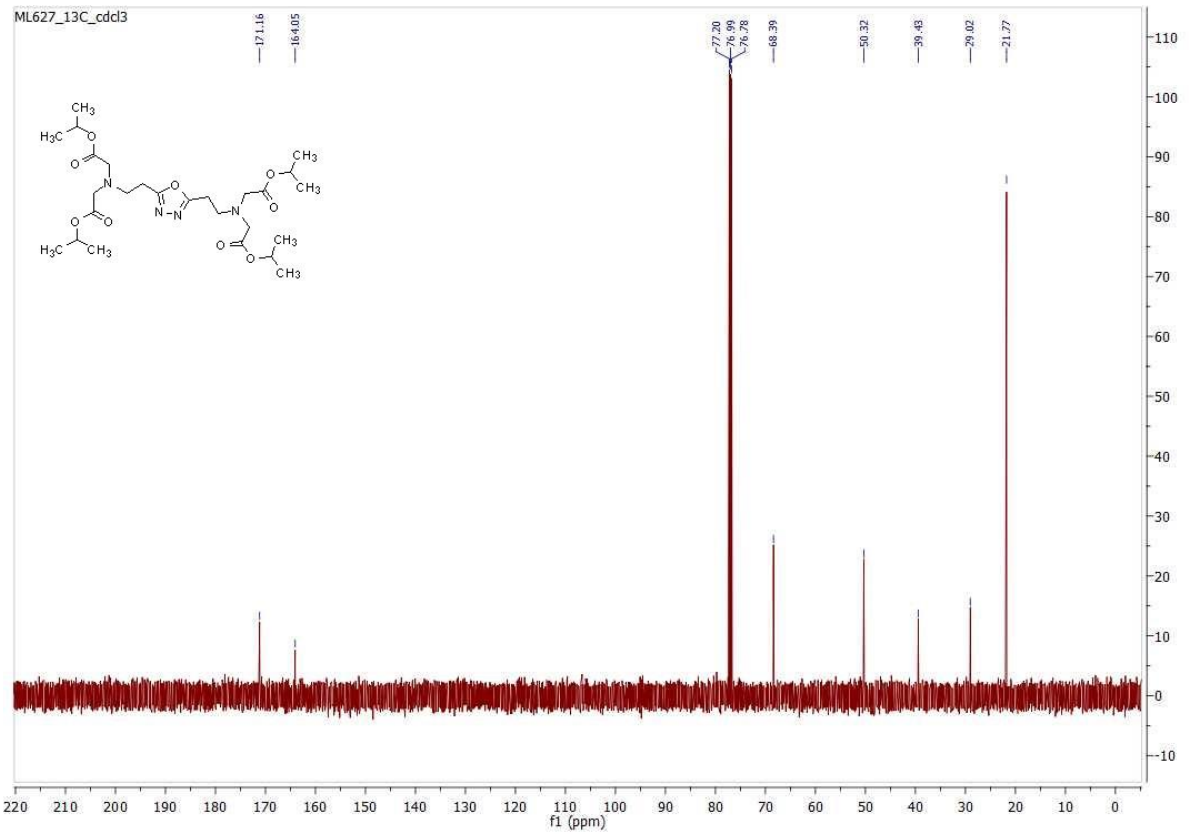

Figure S26. ^{13}C NMR spectra (100 MHz,$CDCl_3$) of Tetraisopropyl 2,2′,2′′,2′′′-(((1,3,4-oxadiazole-2,5-diyl)bis(ethane-2,1-diyl))bis(azanetriyl))tetraacete (**6c**)

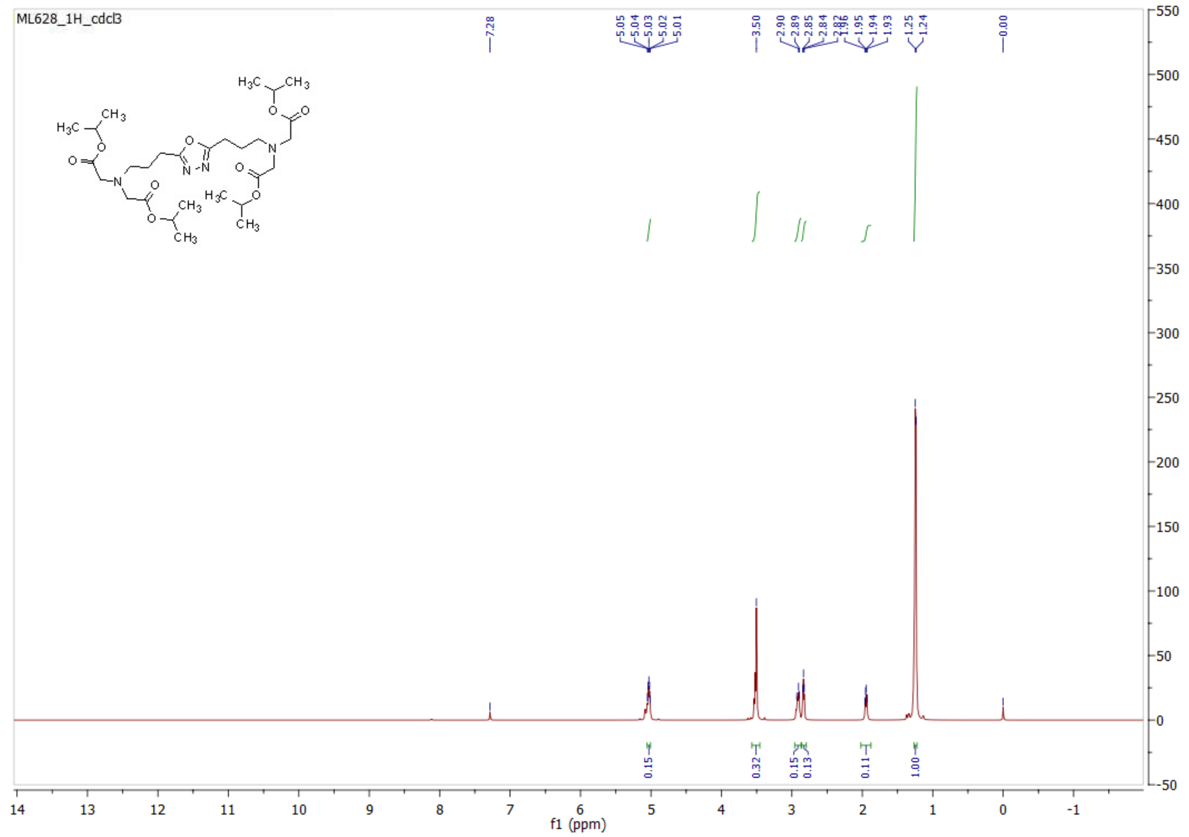

Figure S27. ^{1}H NMR spectra (400 MHz,$CDCl_3$) of Tetraisopropyl 2,2′,2′′,2′′′-(((1,3,4-oxadiazole-2,5-diyl)bis(propane-3,1-diyl))bis(azanetriyl))tetraacetate (**6d**)

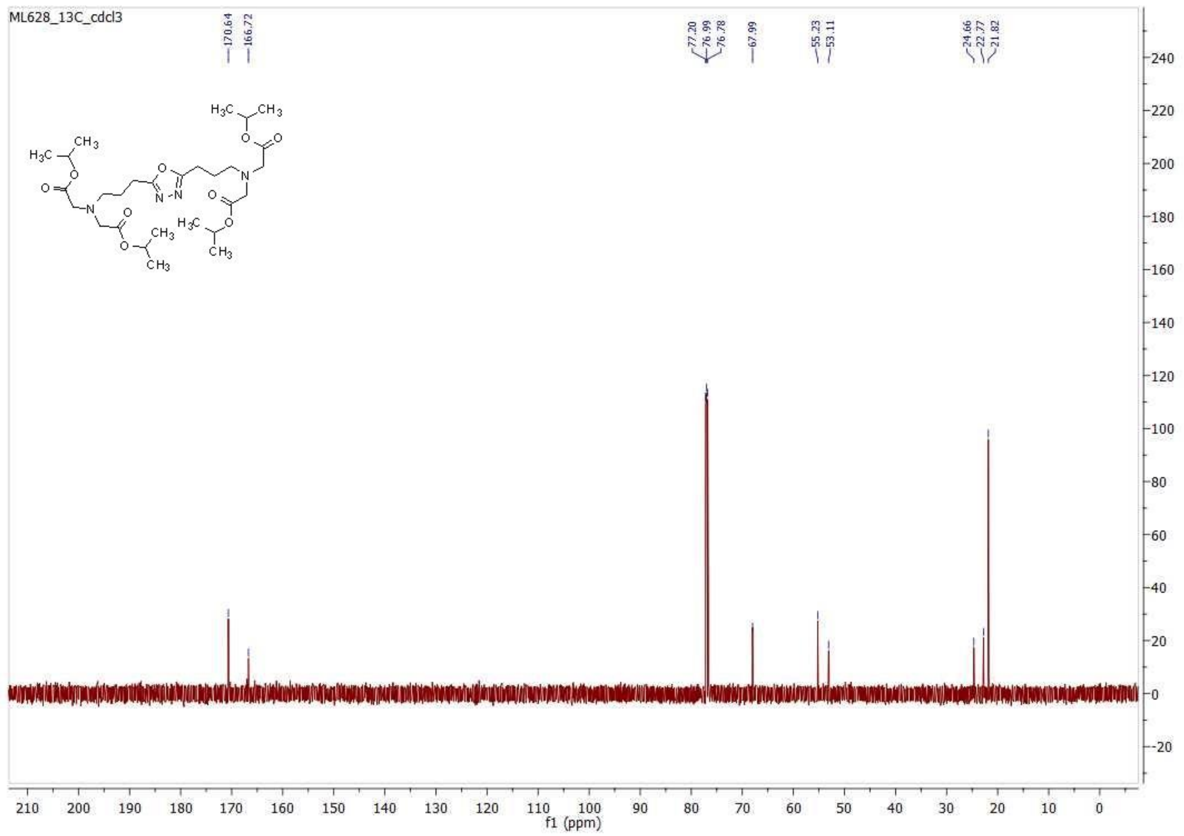

Figure S28. ^{13}C NMR spectra (100 MHz,$CDCl_3$) of Tetraisopropyl 2,2′,2′′,2′′′-(((1,3,4-oxadiazole-2,5-diyl)bis(propane-3,1-diyl))bis(azanetriyl))tetraacetate (**6d**)

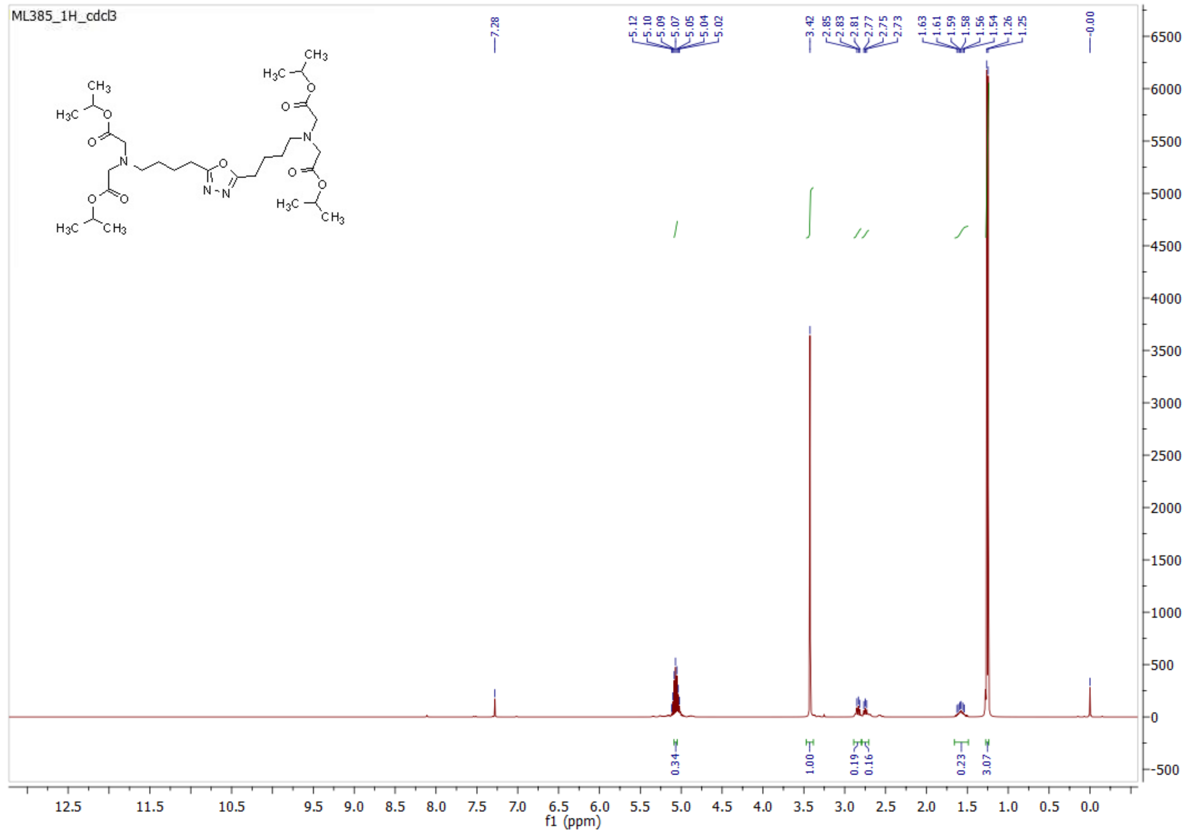

Figure S29. ^{1}H NMR spectra (400 MHz,$CDCl_3$) of Tetraisopropyl 2,2′,2′′,2′′′-(((1,3,4-oxadiazole-2,5-diyl)bis(butane-4,1-diyl))bis(azanetriyl))tetraacetate (**6e**)

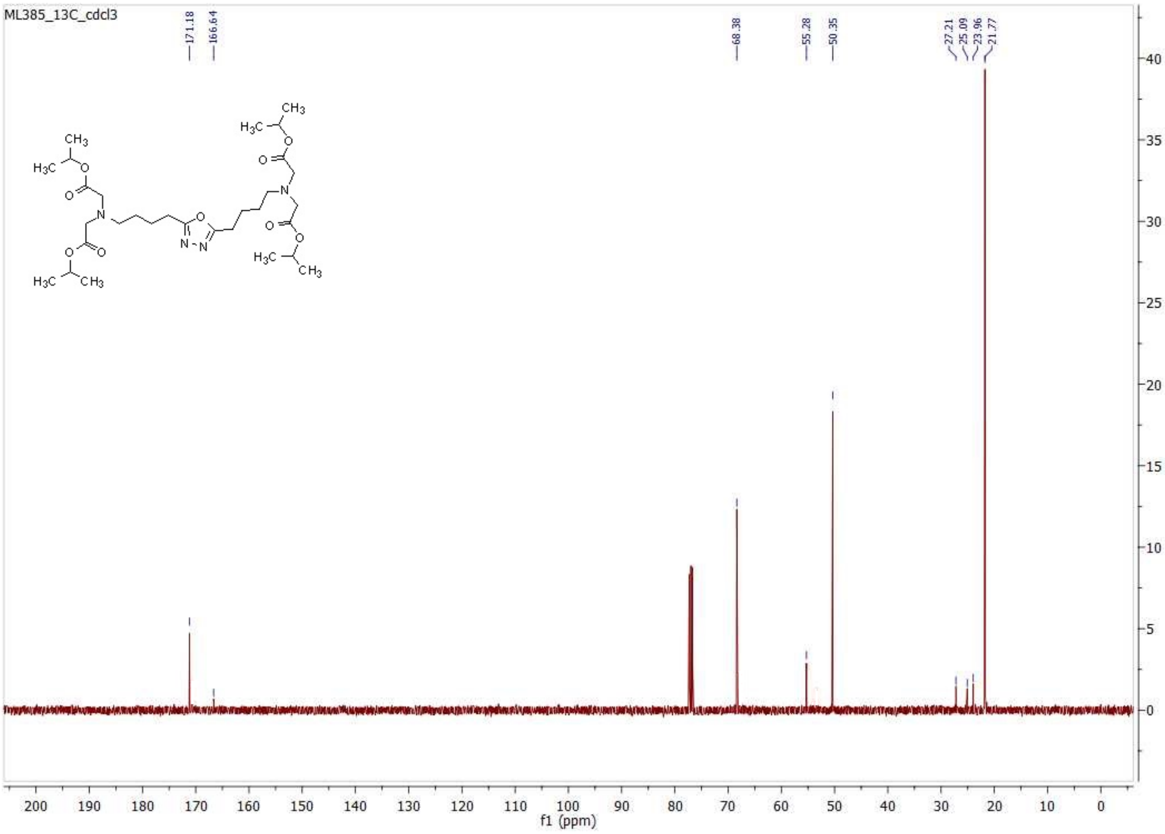

Figure S30. ^{13}C NMR spectra (100 MHz,$CDCl_3$) of Tetraisopropyl 2,2′,2′′,2′′′-(((1,3,4-oxadiazole-2,5-diyl)bis(butane-4,1-diyl))bis(azanetriyl))tetraacetate (**6e**)

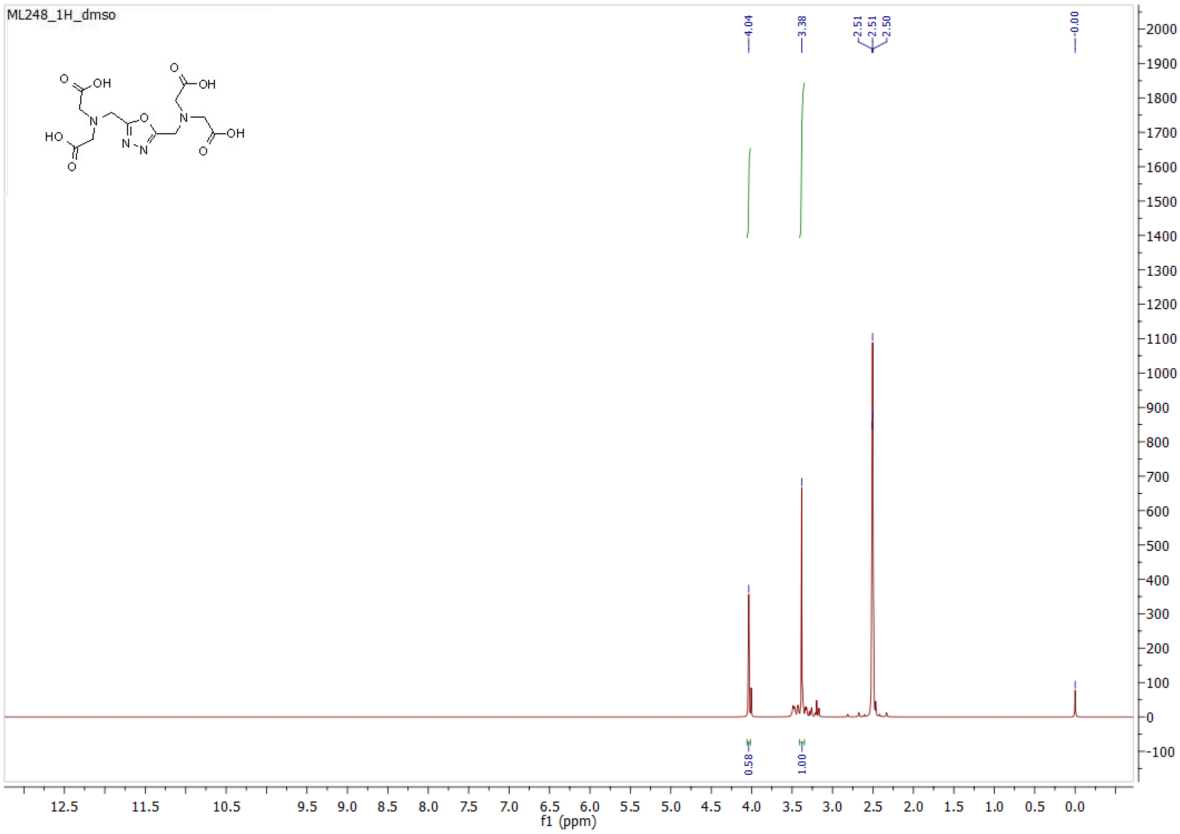

Figure S31. ^{1}H NMR spectra (400 MHz, dmso) of 2,2′,2′′,2′′′-(((1,3,4-oxadiazole-2,5-diyl)bis(methylene))bis(azanetriyl))tetraacetic acid (**7b**)

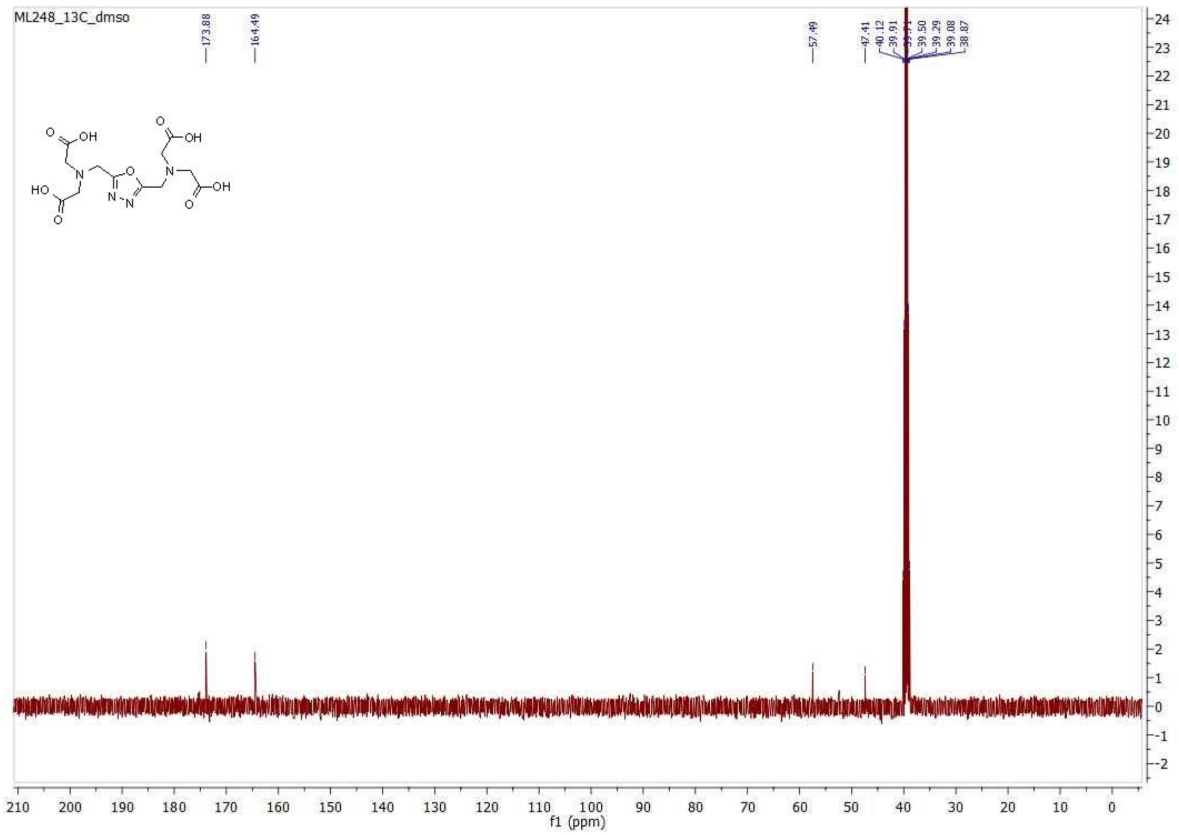

Figure S32. ^{13}C NMR spectra (400 MHz, dmso) of 2,2′,2′′,2′′′-(((1,3,4-oxadiazole-2,5-diyl)bis(methylene))bis(azanetriyl))tetraacetic acid (**7b**)

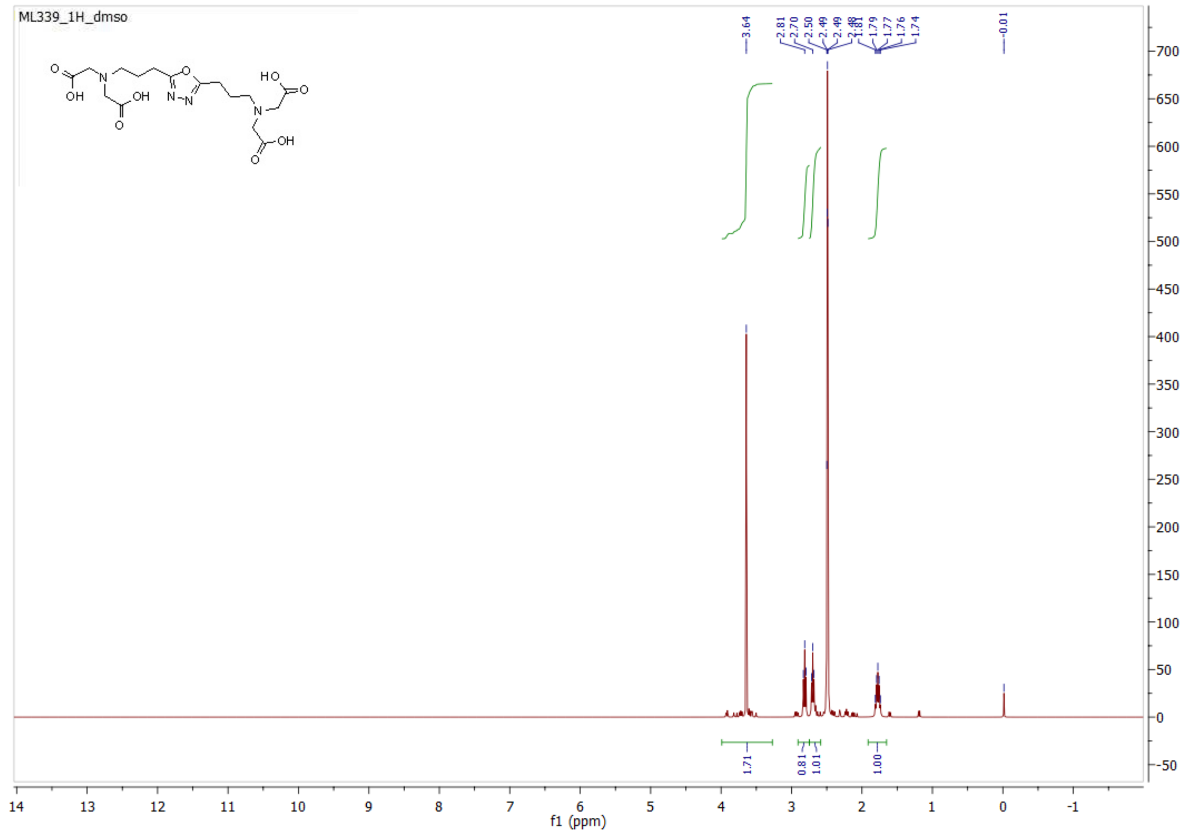

Figure S33. ^{1}H NMR spectra (100 MHz, dmso) of 2,2′,2′′,2′′′-(((1,3,4-oxadiazole-2,5-diyl)bis(propane-3,1-diyl))bis(azanetriyl))tetraacetic acid (**7d**)

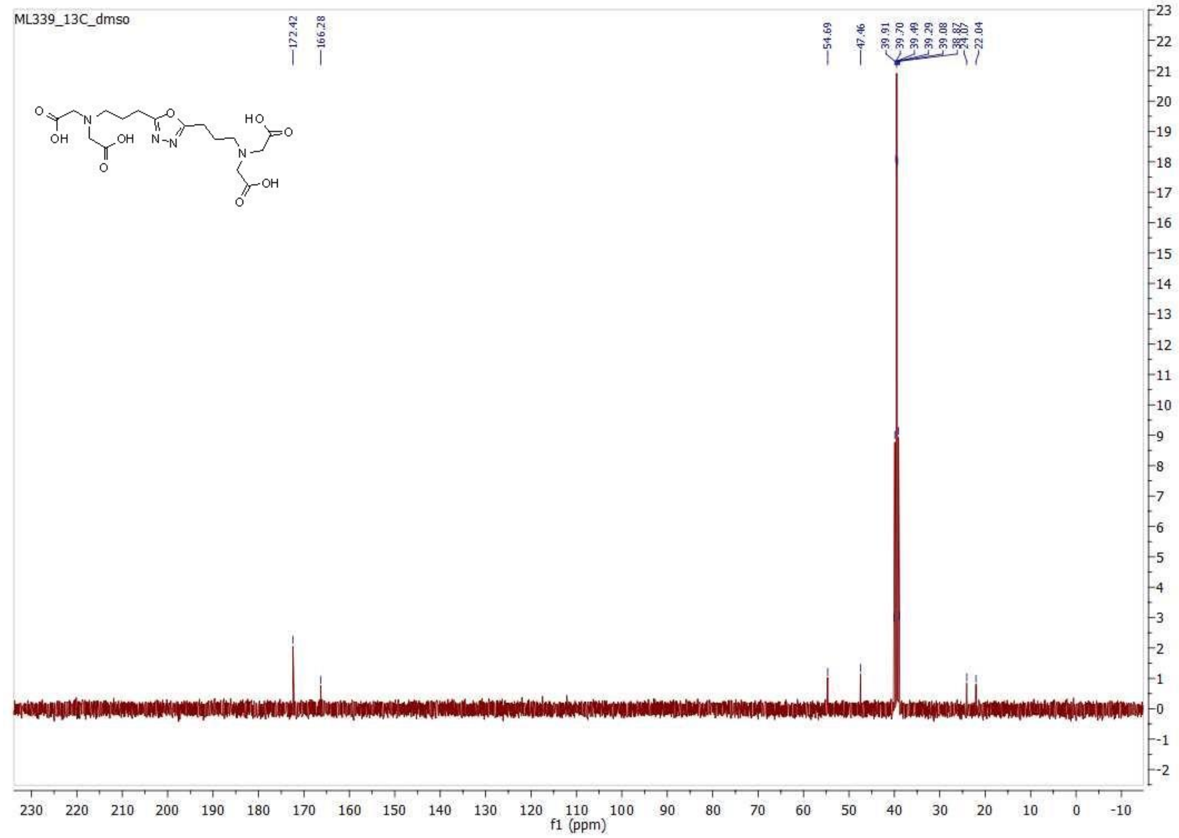

Figure S34. ^{13}C NMR spectra (400 MHz, dmso) of 2,2′,2′′,2′′′-(((1,3,4-oxadiazole-2,5-diyl)bis(propane-3,1-diyl))bis(azanetriyl))tetraacetic acid (**7d**)
